# Formulation of Nano/Micro-Carriers Loaded with an Enriched Extract of Coffee Silverskin: Physicochemical Properties, In Vitro Release Mechanism and In Silico Molecular Modeling

**DOI:** 10.3390/pharmaceutics14010112

**Published:** 2022-01-04

**Authors:** Faezeh Fathi, Samad N. Ebrahimi, João A. V. Prior, Susana M. L. Machado, Reza Mohsenian Kouchaksaraee, M. Beatriz P. P. Oliveira, Rita C. Alves

**Affiliations:** 1 REQUIMTE/LAQV, Laboratory of Bromatology and Hydrology, Department of Chemical Sciences, Faculty of Pharmacy, University of Porto, Rua de Jorge Viterbo Ferreira, 228, 4050-313 Porto, Portugal; FFathi@ff.up.pt (F.F.); smachado@ff.up.pt (S.M.L.M.); remohsenian@gmail.com (R.M.K.); 2 Department of Phytochemistry, Medicinal Plants and Drugs Research Institute, Shahid Beheshti University, Tehran 1983969411, Iran; S_ebrahimi@sbu.ac.ir; 3 REQUIMTE/LAQV, Laboratory of Applied Chemistry, Department of Chemical Sciences, Faculty of Pharmacy, University of Porto, Rua de Jorge Viterbo Ferreira, 228, 4050-313 Porto, Portugal; joaoavp@ff.up.pt

**Keywords:** coffee by-product, phytosome, cholesterol, natural polymer, molecular docking, kinetic release model

## Abstract

Designing strategies for an effective transformation of food waste into high-value products is a priority to address environmental sustainability concerns. Coffee silverskin is the major by-product of the coffee roasting industry, being rich in compounds with health benefits. Such composition gives it the potential to be transformed into high-value products. In this study, coffee silverskin extracts were enriched, regarding caffeine and chlorogenic acid contents, by adsorbent column chromatography. The compounds content increased 3.08- and 2.75-fold, respectively, compared to the original extract. The enriched fractions were loaded into nano-phytosomes or cholesterol-incorporated nano-phytosomes (first coating layers) to improve the physiochemical properties and permeation rate. These nano-lipid carriers were also subjected to a secondary coating with different natural polymers to improve protection and stability against degradation. In parallel, and for comparison, different natural polymers were also used as first coating layers. The produced particles were evaluated regarding product yield, encapsulation efficiency, loading capacity, particle size, surface charge, and in vitro release simulating gastrointestinal conditions. All samples exhibited anionic surface charge. FTIR and molecular docking confirmed interactions between the phytoconstituents and lipid bilayers. The best docking score was observed for 5-caffeoylquinic acid (chlorogenic acid) exhibiting a stronger hydrogen binding to the lipid bilayer. Among several kinetic models tested, the particle release mechanism fitted well with the First-order, Korsmeyer–Peppas, and Higuchi models. Moreover, most of the formulated particles followed the diffusion-Fick law and anomalous transport.

## 1. Introduction

Food processing industries produce relevant amounts of by-products that are discarded, despite their content in valuable nutritional and biological constituents. Their valorization can play a vital role in financial prospects [1]. Therefore, a current concern is to implement the concept of sustainable food waste management worldwide in order to use by-products and convert them into new products of high value, while assuring the development and exploitation of eco-friendly techniques to achieve that goal [2].

Coffee silverskin (SI) is a light brownish pellicle integument of the raw coffee bean. These pellicles are detached from the coffee bean during the industrial roasting process. Given the abundance of coffee factories, SI has become a relevant by-product that should be valorized based on its richness in several phytochemicals, such as caffeine and phenolic compounds [3,4].

Fractionation of plant crude extracts using polymeric adsorbent resin column chromatography promotes an enrichment of bioactive compounds based on functionality and polarity of the phytoconstituents. This enrichment provides an enhancement of the phytoconstituents bioactivity at a low dosage [5].

To overcome limitations related to low stability, absorption rates, bioavailability, or functionality of some phytoconstituents, a wise choice is to focus on natural-based carriers for safe and targeted delivery [6] Accordingly, phytosomes are targeted delivery carriers, characterized by a specific bonding between phospholipids and the compounds of interest (e.g., drugs, phytochemicals, etc.), with possible distinctive configurations. In other words, phytosomes are non-polar carriers for the efficient delivery of bioactive components through the cell membrane. Indeed, they are considered suitable carriers for medicines, dietary supplements, or cosmetic products due to the improved bioavailability and permeation of compounds through the cell membrane, while protecting natural sensitive structures against environmental conditions [7].

Taking into account the low permeation of polar phytoconstituents through the cell membrane, nano-phytosomes based on soybean lecithin (containing 94% phosphatidylcholine) assure significant similarities with the cell membrane to facilitate permeation, a very important requisite for the development of highly efficient delivery carriers [8]. Additionally, the incorporation of cholesterol into the nano-phytosome lipid layer (cholesterol-incorporated nano-phytosomes) by surface modification provides improved stability of the nano-phytosome in solution media [9]. 

Natural polymers (NP), which are non-toxic, biodegradable, and biocompatible, can also protect phytoconstituents or drugs, through encapsulation techniques, improving stability during storage and handling, and also allowing a controlled release [10]. Therefore, NP coating of extracts or nano-phytosomes (being, in this last case, a secondary coating) in the form of small capsules obtained by spray-drying can also be applied to enhance protection, solubility, and stability of the compounds in acidic media, and to improve shelf-life during handling and storage [11]. 

The main aim of this work was to formulate and compare different types of nano/micro-carriers loaded with an SI extract enriched in caffeine and phenolic compounds with antioxidant properties. A potential application, for instance, in the dietary supplement field is foreseen, more specifically to improve mental and physical performance, competing with other products already existing in the market [12]. A final product is expected that is well absorbed and tolerated, which efficiently delivers the mentioned phytochemicals in targeted body areas, to more effective bioactivity at lower dosages. 

For that, lipid carriers (nano-phytosomes or cholesterol-incorporated nano-phytosomes) were applied as first layers to an SI-enriched extract, in order to enhance the phytochemicals permeation rates. In parallel, and for comparison, different NPs were also used as first layers, directly coating the SI-enriched extract (simple encapsulation). Additionally, both lipid-based layers (nano-phytosomes and cholesterol-incorporated nano-phytosomes) were also coated with different NPs as secondary coating layers to increase stability during storage and handling, improve adhesion affinity of particles (which enhance phytochemicals uptake), and provide a controlled release [13,14,15]. Afterwards, the in silico molecular docking was used to confirm the molecular arrangement and interactions among the lipid bilayer and specific phytochemicals [16]. Indeed, this study presents, for the first time, by molecular docking, the interaction between the nano-phytosome bilayer and two major constituents of SI, namely, caffeine and chlorogenic acid (5-caffeoylquinic acid). The arrangement of other phytochemicals within similar lipid bilayers was also recently reported for the first time by our research group [16].

## 2. Materials and Methods

### 2.1. Chemicals and Standards

Gallic acid (CAS 149-91-7) and chlorogenic acid 95% (CAS 327-97-9) were supplied by Sigma, Life Science, Shanghai, China. Apple pectin (CAS 9000-09-5) was purchased from LABChem, Lisbon, Portugal; maltodextrin (CAS 32671-12) from Fargon, Barcelona, Spain; and gum Arabic from Guinama, Valencia, Spain. Water-soluble starch from potato (CAS 9005-84-9) was supplied from PanReac-Applichem, Darmstadt, Germany. Soybean lecithin (CAS 8002-43-5; purity of 90%) was obtained from Alfa Aesar, Thermofisher, Kandel, Germany. Cholesterol (CAS C8667-25G; purity ≥ 99%) was supplied by Sigma, Life Science, St. Louis, MO, USA. Absolute ethanol (≥99.8% and 96% purity), dichloromethane (≥99.9%), and HPLC grade analytical solvents were purchased from Honeywell (Hessen, Germany). Relite EXA90 (ion exchange resin) Diaion^TM^ HP20LX (spherical porous polystyrene resin) were both kindly supplied by the Resindion group of Mitsubishi Chemical, Binasco, Italy. Sodium phosphate dibasic heptahydrate (Na_2_HPO_4_·7H_2_O) and sodium phosphate monobasic monohydrate (NaH_2_PO_4_·H_2_O) were purchased from PanReac, Barcelona, Spain. Ultra-pure water was obtained from Seralpur PRO 60 CN and Seradest LFM 20 water purification systems.

### 2.2. Samples

SI was kindly provided by a Portuguese coffee roaster industry (BICAFÉ). It resulted from the roast of commercial batches composed of both *Coffee arabica* and *Coffea canephora* beans and was representative of the major by-product of the factory.

### 2.3. Experimental Design

**Chart 1:**
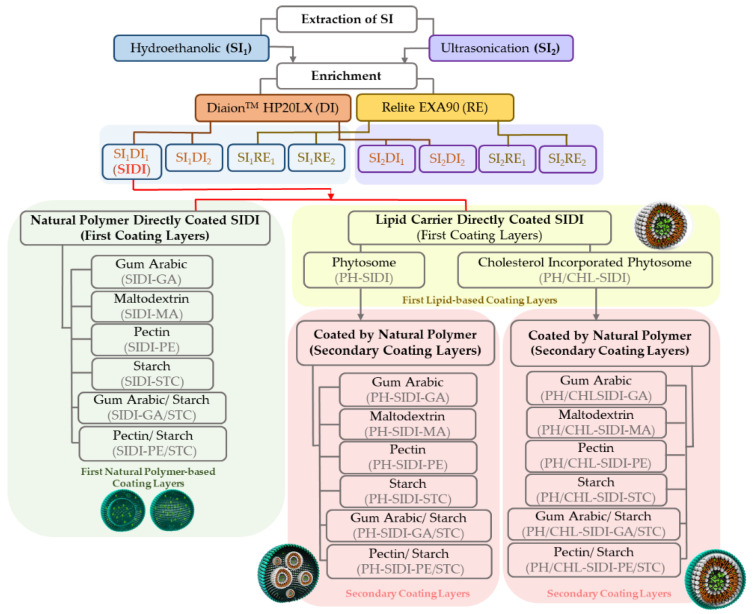
Flow of the preparation steps, regarding the type of extraction, enrichment, and type of coating layer (first or secondary layers). SI, silverskin; DI, Diaion resin; RE, Relite resin; GA, gum Arabic; MA, maltodextrin; PE, pectin; STC, starch; PH, nano-phytosome; PH/CHL, cholesterol incorporated nano-phytosome. Note: DI_1_ and RE_1_ series are the enriched fractions (regarding caffeine and phenolics contents) obtained with DI and RE, respectively. DI_2_ and RE_2_ series are the discarded fractions (rich in sugars, chlorophylls, etc.).

Chart 1 aims to clearly introduce the flow of the preparation steps, regarding the type of extraction, enrichment, and type of coating layer in terms of first and secondary layers. The final product codes for each category are also presented in Chart 1.

**Chart 2:**
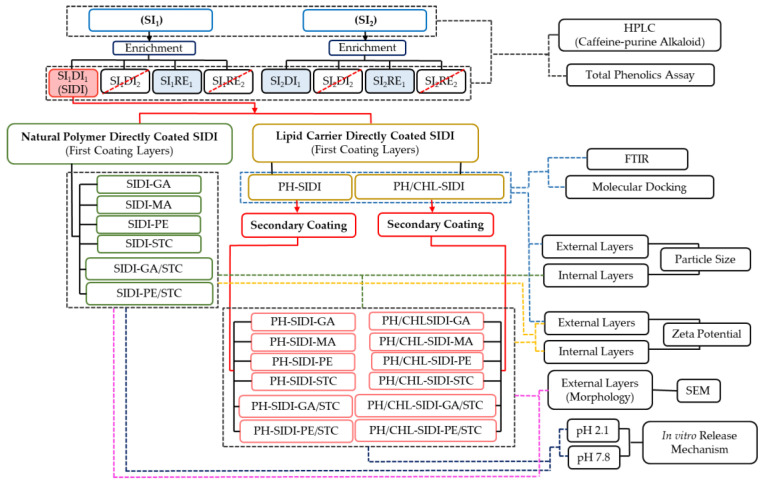
Sample categorization and analyses applied to each category. SI, silverskin; GA, gum Arabic; MA, maltodextrin; PE, pectin; STC, starch; PH, nano-phytosome; PH/CHL, cholesterol incorporated nano-phytosome. DI_1_ and RE_1_ series are the enriched fractions (regarding caffeine and phenolics contents) obtained with Diaion^TM^ HP20LX (DI) and Relite EXA90 (RE) adsorbent resins, respectively. DI_2_ and RE_2_ series are the discarded fractions (rich in sugars, chlorophylls, etc.).

SI was extracted using different techniques (maceration with a hydroethanolic solvent (SI_1_) or ultrasonication (SI_2_)) and each extract was enriched (to increase caffeine and phenolics contents) using one of these resins: Diaion^TM^ HP20LX (DI) or Relite EXA90 (RE). DI_1_ and RE_1_ series are the enriched fractions obtained with DI and RE, respectively. DI_2_ and RE_2_ series are the rejected fractions (explained in Section 2.4. and Chart 2)

The most enriched fraction (SI_1_DI_1_) was then selected for the following formulation procedures. SI_1_DI_1_ (from now on named SIDI) was loaded into nano-phytosomes or cholesterol-incorporated nano-phytosomes as first coating layers (Chart 1, in yellow). In parallel, SIDI was also directly coated with NP as the first coating layer (single coating layer) (Chart 1, in green). Meanwhile, to improve the stability and protection of lipid-based carriers, NPs were also used to prepare secondary layers, encapsulating the abovementioned nano-phytosomes and cholesterol-incorporated nano-phytosomes (Chart 1, in pink). In sum, two types of the first coating layers were prepared, namely NP-based and lipid-based. In addition, these lipid-based layers (nano-phytosomes and cholesterol-incorporated nano-phytosomes) were also coated with different NPs as the secondary layer.

### 2.4. Preparation of SI Extracts

SI was extracted using two different methodologies: A hydroethanolic maceration (SI_1_) and an ultrasonication extraction (SI_2_). 

The hydroethanolic maceration used 100 g of SI and occurred in a 5 L glass flask during 24 h for each solvent. It was performed in triplicate, with different and subsequent ethanol:water mixtures (100:0, 50:50, 50:70, and 0:100; 2 L each). A filtration step followed each maceration. The four extracts were combined, and ethanol was recovered using a rotating vacuum evaporator at 40 °C. The concentrated aqueous extract (SI_1_) was stored at 2 °C prior to the next step.

Ultrasonic extraction was performed, in triplicate, based on the procedure described previously by Puga et al. [17] with some modifications. The sample (100 g) was macerated for 2 h in 2 L of deionized water, and sonicated with an ultrasonic probe (BANDELIN electronic, UW 50, Berlin, Germany) during 30 min, at 25 °C. After, the solutions were filtered and concentrated using a rotating vacuum evaporator at 40 °C. The concentrated aqueous extract (SI_2_) was stored at 4 °C prior to the next step.

The enrichment was designed to concentrate SI_1_ and SI_2_ regarding the contents of caffeine and total phenolics, and was performed according to the methodology described and scaled up at the Medicinal Plants and Drugs Research Institute—Shahid Beheshti University [5]. Diaion^TM^ HP20LX (DI) and Relite EXA90 (RE) adsorbent resin columns (50 cm × 6 cm) were used separately, according to the procedure described in Chart 1. Both columns were activated with ethanol ≥ 99.8% for 12 h, followed by solvent removal and washing with 5 L of distilled water. A 6 g amount of the concentrated aqueous extracts (SI_1_ and SI_2_) were diluted in distilled water (500 mL) and loaded into the resin columns at a flow rate of 5 mL/min and kept for 30 min for bioactive compounds (caffeine and phenolics) adsorption in resin porous. A new washing of the resin columns, with 5 L of distilled water, was performed to remove residues (sugar, chlorophyll, etc.). These fractions (SI_1_DI_2_, SI_2_DI_2_, SI_1_RE_2_, and SI_2_RE_2_) were discarded (Chart 2). Afterwards, the columns were eluted with ethanol ≥ 99.8% (2 L) for the desorption of caffeine and phenolics. Ethanol was recovered using a rotating vacuum evaporator and the enriched fractions were frozen at −80 °C, and lyophilized (48 h, −80 °C, 0.022 mbar; TELSTAR, Cryodos freeze dryer, Barcelona, Spain). These lyophilized enriched fractions (SI_1_DI_1_, SI_1_RE_1_, SI_2_DI_1_, SI_2_RE_1_) were kept in the refrigerator at 2–4 °C for further analysis and to follow the next steps in accordance with Chart 1 and Chart 2) [18,19].

### 2.5. Phytochemicals Analyses

#### 2.5.1. Caffeine Analysis by High-Performance Liquid Chromatography (HPLC)

The caffeine content of the extracts was analyzed using an HPLC-DAD system (Jasco, Tokyo, Japan). This system consisted of an LC-NetII/ADC hardware interface, an automatic sampler (Jasco AS-2057 Plus), a pump (Jasco PU-2089 Plus), a multi-wavelength diode array detector (DAD, Jasco MD-2018 Plus), and a column oven (Jasco CO-2060 Plus). The gradient elution used was the following: 0 min, 5% B; 40 min, 25% B; 55 min, 45% B; 60 min, 60% B; 65 min, 5% B (solvent A: 0.5% acetic acid; solvent B: 100% methanol), with a flow rate of 1.1 mL/min. The chromatographic column was a Zorbax-SB-C18 (5 μm, 250 mm × 4.6; Agilent Technologies, Santa Clara, CA, USA), at 28 °C. The DAD recorded data from 200 to 600 nm were monitored at 274 nm. For HPLC analyses, the lyophilized extracts were dissolved in H_2_O (10 mg/mL) and the injected volume was 20 µL. Caffeine was used as the standard for HPLC analyses validation. The calibration curve (y = 36,096x − 227,800; R^2^ = 0.9996) was constructed in the linear range of 1.5–800 µg/mL. The detection limit of the method was 1.24 µg/mL.

#### 2.5.2. Total Phenolics Assay

Total phenolics contents were determined as described by Costa et al. [3]. Briefly, 150 µL of the Folin–Ciocalteu reagent (1:10) and 120 µL of a sodium carbonate solution (7.5% *m/v*) were added to 30 µL of the extract, followed by incubation at 45 °C for 15 min and 30 min at room temperature. Absorbance was monitored at 765 nm (BioTek, Synergy HT, PMT 49,984, Winooski, VT, USA). Two calibration curves were prepared using gallic acid (5–100 µg/mL; y = 0.0085x −0.0544; R^2^ = 0.998) and chlorogenic acid (5–140 µg/mL; y = 0.0056x − 0.0364; R^2^ = 0.9973) as standards.

### 2.6. Particles Preparation and Characterization

#### 2.6.1. Preparation of First Coating Layers: Coating Layers Based on Natural Polymers

Gum Arabic (GA), maltodextrin (MA), pectin (PE), and starch (STC), individually, and blends (1:1) of pectin/starch (PE/STC) and gum Arabic/starch (GA/STC) were used as the first NP coating layers (Chart 1, Table 1). The feed solution had 10% (*w*/*v*) of NP and 1% (*w*/*v*) of SIDI (selected as the best enriched extract, Chart 1). These proportions were used because they do not compromise the layer efficiency and have an adjusted viscosity for feeding the spray dryer. In this context, six different NP-based solutions were prepared in 100 mL of distilled water. The prepared solutions were stirred and homogenized for 2 h at 55 °C and, afterwards, injected into the spray dryer under specific conditions as described in Section 2.6.5.

#### 2.6.2. Preparation of First Coating Layers Based on Lipids: Nano-Phytosomes

Nano-phytosomes were produced by the thin film hydration method described by Fathi and Ebrahimi, with small modifications [5]. To decrease the size of the obtained particles, in the hydration step, a sonication probe was applied. Phytosomes were prepared with lecithin (containing 94% phosphatidylcholine) and loaded with the SIDI enriched fraction, with an optimum molar ratio of (1:1) (Table 1 and Chart 1). This lecithin-based layer was another type of the first coating layer (phytosome coating layer). The experimental procedures were performed as follows: 50 mg of SIDI dissolved in 20 mL of ethanol was placed in a 50 mL round flask and heated up to 55 °C until a transparent solution was achieved. Then, 50 mg of lecithin was dissolved in 2 mL of dichloromethane and vortexed (Shaker & Mixers Reax top, Heidolph, Schwabach, Germany) for 5 min, at room temperature, to obtain a transparent yellow solution. Afterwards, the lecithin solution in dichloromethane was sprayed on the SIDI ethanolic solution and refluxed under stirring for 2 h at 55 °C. The solution was cooled, and the solvent evaporated (rotary vacuum evaporator) at 37 °C at a medium speed from 40 to 200 rpm and 55 mbar of vacuum pressure until a thin layer was shaped. Afterwards, N_2_ was flushed on the thin layer for 1 min, the flask was sealed, and kept overnight on a desiccator [20]. Thereupon, the phytosome mother solution (PH-SIDI) was formed by hydration of the thin film layer with phosphate buffer (pH 5.5), at 40 °C for 10 min (vacuum pressure: 200 mbar; rotation: 200 rpm). Afterwards, ultra-sonication was applied for 25 min, at 60% amplitude in pulsation mode (5:1 s) (total energy: 10,000 ± 100 kJ) [21].

#### 2.6.3. Preparation of First Coating Layers Based on Lipids: Nano-Phytosomes Incorporated by Cholesterol 

Cholesterol-incorporated nano-phytosomes consist of a combination of SIDI, lecithin, and cholesterol in an optimum molar ratio (1:1:0.5) treated according to the description in Section 2.6.2, with some modifications. This was the third type of the first coating layer developed in this study.

The combination of SIDI and lecithin was performed as described in the previous section (Section 2.6.2) and stirred for 10 min (Solution A). Afterwards, 25 mg of cholesterol was dissolved in 2 mL of dichloromethane and vortexed for 5 min. This solution was sprayed on solution A and refluxed under stirring for 2 h at 55 °C. The solvent was evaporated using a rotary vacuum evaporator at 37 °C, at a medium speed of 40 to 200 rpm, and a vacuum pressure of 55 mbar, until a thin layer was shaped. After, N_2_ was flushed on the thin layer for 1 min, and the flask was kept overnight in the desiccator. The solution of cholesterol-incorporated nano-phytosomes loaded with SIDI (PH/CHL-SIDI) was hydrated using the same conditions described in the previous section (Section 2.6.2) with phosphate buffer (pH 7.8) (Table 1).

#### 2.6.4. Preparation of Secondary Coating Layers (Coating of Nano-Phytosomes and Cholesterol-Incorporated Nano-Phytosomes with Natural Polymers as Secondary Layers)

The secondary layer was prepared with 10% (*w*/*v*) of NP for PH-SIDI, and 15% (*w*/*v*) of NP for PH/CHL-SIDI. For this purpose, immediately after preparation, the lipid coating layers (PH-SIDI or PH/CHL-SIDI), the solutions of NP in the aforementioned concentration, were individually added (Table 1). The NP were first dissolved in ultrapure water and stirred for 2 h at 55 °C. After, the NP solution was mixed and added to PH-SIDI or PH/CHL-SIDI (Table 1, Chart 1) and subjected to ultrasonication with a probe for 5 min, in pulsation mode (5:1 s) and stirred for 20 min. The solutions were then injected into the spray dryer to obtain the final powder (Table 1 and Chart 1) [16].

#### 2.6.5. Spray Drying Conditions for Encapsulation

A mini spray-dryer B-290 BÜCHI (Flawil, Switzerland) with a standard nozzle (0.5 mm) was used for the drying of NP coating and secondary NP coating layers. The encapsulation procedure was optimized based on previously reported data with some modifications [16]. The different prepared emulsions (Section 2.6.1 and Section 2.6.4) fed the spray dryer under optimized stirring conditions, at a flow rate of 10 mL/min, aspiration of 100% (36 m^3^/h), air pressure of 5.5–6 bar, and a nozzle cleaner set to 3. Although all solutions were prepared in ultrapure water, the drying process used the same drying conditions with different drying temperatures. The inlet temperature differed according to the type of the NPs. In the case of starch, gum Arabic/starch, and pectin/starch, the drying temperatures were 135 °C as the inlet temperature and 63 ± 3 °C as the outlet temperature. In the case of pectin, the inlet temperature was set at 120 °C, and the outlet temperature almost 58 ± 2 °C. For the other NP, the inlet temperature was set at 115 °C, and the outlet temperature at 55 ± 2 °C. Throughout the injection process, a magnetic stirrer, at room temperature, shook the emulsions continuously to avoid aggregation of its solid content. Finally, the particles were recovered from the collector, sealed in aluminum foil, and stored at 4 °C for further analyses. The product yield (%) was calculated by the amount of the particles recovered from the drying step divided by the total mass content of the initial feeding solution (Equation (1)) [22,23].

(1) $$Yeild\;\left(\%\right)=\frac{Total\;weight\;of\;polymeric\;or\;binary\;layers\;in\;powder\;\left(mg\right)}{Total\;weight\;of\;feed\;solution\;\left(combination\;of\;all\;ingridient\right)\;\left(mg\right)}\times 100$$

### 2.7. Physicochemical Properties of Nano/Micro-Particles

#### 2.7.1. Encapsulation Efficiency and Loading Capacity

The encapsulation efficiency (EE) was defined by the SIDI concentration successfully entrapped in the particles. The EE% is the amount of drug successfully trapped in the particles (the total drug added subtracted by the non-entrapped drug) divided by the total quantity of the drug initially added in preparation steps [24,25]. Therefore, the encapsulation efficiency expressed in a percentage (EE%) and the loading capacity (LC) were calculated using the amount of the remaining free caffeine in the surface of the powder particles quantified by HPLC-DAD immediately after the preparation steps, according to the chromatographic conditions described in Section 2.5.1.

For that, 10 mg of particles were dissolved in 1 mL of solvent (ultrapure water and ethanol 30:70, *v*/*v*) under continuous stirring for 10 min, followed by centrifugation (HERAEUS FRESCO 17, Thermo Fisher Scientific, LR56495, Bremen, Germany) for 6 min, at 12,000 rpm. After, a 0.45 μm pore size PTFE syringe filter was used to filter the samples before HPLC injection.

The EE% was calculated via Equation (2). This method was previously described [24,25]. Regarding Equation (2), the amount of encapsulated caffeine in the solution is the caffeine_experimental_ (calculated using HPLC data), and the caffeine_theoretical_ is the SIDI total amount of caffeine hypothetically present in the particles.

(2) $$\mathrm{EE}\left(\%\right)=\frac{{\mathrm{caffeine}}_{\mathrm{experimental}}}{{\mathrm{caffeine}}_{\mathrm{theoretical}}}\times 100$$

Thereafter, taking into consideration Equation (3), theoretical LC is the caffeine content if 100% of SIDI caffeine were trapped in the particles. The specific LC% was calculated using Equation (3) [24].

(3) $$\mathrm{LC}\;\left(\%\right)=\mathrm{EE}\times {\mathrm{LC}}_{\mathrm{theoretical}}\times 100$$

#### 2.7.2. Fourier-Transform Infrared Spectroscopy Analyses (FTIR)

The interaction between lecithin and cholesterol with SIDI in the phytosomes (PH-SIDI) and cholesterol-incorporated phytosomes (PH/CHL-SIDI) in a lyophilized form (dried with TELSTAR freeze dryer, Cryodos, Spain) were evaluated using a Fourier transform infrared (FTIR) apparatus (Frontier, PerkinElmer, Beaconsfield, UK) equipped with an attenuated total reflectance (ATR) accessory (PerkinElmer, Beaconsfield, UK), operated by spectrum software (PerkinElmer, Beaconsfield, UK).

#### 2.7.3. Particle Size Distribution and Zeta Potential (Surface Charge)

Particle size distribution was assessed using a particle size analyzer (Brookhaven Instruments Corporation, operated by particle sizing v.5 Brookhaven instruments software, Holtsville, NY, USA). The qualitative particle properties were determined in PH-SIDI and PH/CHL-SIDI in solution, in external and internal NP coating and NP-secondary coating I and II layers in powder. The dried particles were dispersed in ethanol 99% to avoid probable agglomeration and sonicated (SOLTEC, SONICA 2200MH S360Hz, Milano, Italy) for 7 min to eliminate the agglomeration and deformation of external layer. In the case of PH-SIDI and PH/CHL-SIDI solutions, the small droplets were dispersed in ethanol 99% and sonicated for more 7 min to avoid aggregation of lipid colloids. The particle size was characterized by mean size in volume and number, obtained in six runs of 1 min at 21 °C. The particles surface charges were obtained with ZetaPLAS (Zeta Potential Analyzer, Brookhaven Instruments Corporation, operated by the PALS Zeta Potential Analyzer v.5 Brookhaven Instruments software, Holtsville, NY, USA), after 6 runs of 30 s at 21 °C. All samples were analyzed regarding individual parameters in separated runs.

#### 2.7.4. Scanning Electron Microscopy (SEM)

The study of the size and surface morphology of polymeric and secondary layers (external layer) used surface structural analysis under images performed by SEM (Fei Quanta 400 FEG ESEM/EDAX Pegasus X4M). Beforehand, samples were adjusted on a brass stub (carbon stub) using double-sided adhesive tape, dried under a N_2_ stream, and then coated by electrical conductivity (a thin layer of gold) in a vacuum by sputtering in a JEOL JFC 100 apparatus at Centro de Materiais da Universidade do Porto (CEMUP).

#### 2.7.5. In Vitro Drug Release

The in vitro drug release study (dissolution assay) was based on the caffeine release from particles over time, assessed in a gastrointestinal simulator containing 100 mL PBS buffer (pH 7.8 or 2.1), a Spectra/Por^®^ Dialysis membrane standard (RC Tubing, MWCO: 3.5 KD, width: 45 mm, diameter: 29 mm, USA & Canada), with a stirrer speed of 70 rpm and heated at 37 ± 2 °C. The media was sealed and protected to avoid evaporation during the process. For that, 10 mg of powder was dispersed in ethanol 99% and placed inside the dialysis tubing. The end of the dialysis membrane was closed with clamps, placed horizontally in the middle of PBS media, and the top was stuck with wires. A caffeine calibration curve (2.5–120 µg/L; y = 0.0051 × +0.1038) was used to calculate the total amount of drug release. “t” (time) corresponds to the presence of the characteristic peak of caffeine, evaluated by UV-Vis spectrometry at a maximum wavelength of 274 nm (BioTek, Synergy HT, PMT 49984, USA). The caffeine release was monitored from time zero to 72 h, at regular time intervals, based on the release route of each sample, in triplicate [24]. 

#### 2.7.6. Mathematical Models of Kinetic Release

Mathematical modeling was exploited to adjust the results obtained in Section 2.7.5. In vitro drug release to different kinetic release models was used as a tool to obtain critical parameters that allow one to predict important aspects concerning the release/dissolution profile. The application of the kinetic model to predict the release behavior of phytoconstituents/drugs was previously reported [26,27,28]. Therefore, caffeine release from the synthesized particles was evaluated through correlation studies between the caffeine release data into the medium with different pHs and mathematical kinetic models, namely, zero-order (Equation (4)), first order (Equation (5)), Korsmeyer–Peppas (Equation (6)), Higuchi (Equation (7)), and Hixson–Crowell (Equation (8)) [29,30], as described below:

(4) $$F_{t}=F_{0}+K_{0}t$$

where “F_t_” is the cumulative amount of the active ingredient released at the time “t”, “F_0_” is the initial amount of the active compound in solution (normally F_0_ = 0), “K_0_” is the zero-order release constant, and “t” is a time value.

(5) $$F_{t}=\mathrm{Fmax}\times \left(1-e^{K_{1}t}\right)$$

where “F_t_” is the cumulative amount of the active ingredient released at the time “t”, “F_max_” is the maximum cumulative amount, and “K_1_” is the first-order release constant.

(6) FtF∞=FKP tn

where “F_t_/F_∞_” is the amount of active compound released until time “t”, “F_KP_” is the Korsmeyer–Peppas constant, “t” is a value of time, and n (release exponent) is an estimating different release mechanism. When *n* < 0.43, drug transport (mass transfer) occurs by pure diffusion following the Fick law (case-I transport). In the case of *n* > 0.43, the mass transfer follows a non-Fickian model; when 0.43 < *n* < 0.85, drug transport occurs based on anomalous drug transport resulting in a combination of Fickian diffusion and swelling release (case II). If *n* is equal to 0.85, drug transport occurs based on Case-II (zero-order kinetic controlling swelling and relaxation of polymer matrix). Finally, when *n* > 0.85, drug transport occurs based on super case-II transport [29,31].

(7) $$F_{t}=F_{H}\;\sqrt{t}$$

where “F_t_” is the cumulative amount of the active ingredient released at the time “t”, “F_H_” is the Higuchi constant, and “t“ is a value of time.

(8) $$F_{t}=100\times \left[1-{\left(1-K_{\mathrm{HC}}\times t\right)}^{3}\right]$$

where “F_t_” is the cumulative amount of the active ingredient released at the time “t”, “K_HC_” is the Higuchi constant, and “t” is a value of time.

The fitting of a kinetic model to a release profile is dependent on the calculated adjusted correlation coefficient (R^2^_adj_), since the correlation coefficient (R^2^) is influenced by the number of parameters in the equation, increasing with the number of those parameters [32,33,34].

#### 2.7.7. Molecular Docking Arrangement

The 3D structures of phosphatidylcholine (≥ 94% of lecithin), cholesterol, and the two main phytochemicals of SI—caffeine and 5-caffeoylquinic acid (chlorogenic acid)—were downloaded from the ChemSpider database. They were prepared and refined using the Ligprep application (Maestro 12.8, Schrödinger, New York, NY, USA). The 3D models of nano-phytosome and cholesterol-incorporated nano-phytosome bilayers were generated using a MemGen web server defined for lipid membrane simulation systems [35]. The generated models were downloaded in PDB format and subjected to additional optimization using the OPLS3 force field using Maestro 12.8. To investigate the interaction of the phytochemicals with the related bilayers, a grid box (x = 8.96, y = 28.10, z = 30.36, Size of 40, 40, 40 Å) was created by grid generation. The interaction between ligands and bilayers was carried out using a glide application with extra precision (XP) level in Maestro 12.8. For each ligand, five poses have been used to evaluate docking interactions.

### 2.8. Statistical Analysis

The results were expressed as mean ± standard deviation. A One-way ANOVA test followed by post-hoc comparisons with Tukey’s HSD was used to identify significant differences between samples at *p* < 0.05 (IBM SPSS 25 for Windows, IBM Corp., Armonk, NY, USA).

## 3. Results

### 3.1. Phytochemical Profiling and Enrichment Efficiency

SI was extracted with the hydroethanolic solvent (SI_1_) and ultrasonication with water (SI_2_). The extracts were enriched using Diaion^TM^ HP20LX (DI) and Relite EXA90 (RE) adsorbent resins (Chart 1) regarding the caffeine and total phenolics contents.

The highest extraction yield was achieved with the hydroethanolic extraction (SI_1_, 66.58%), being almost twice that of those obtained with ultrasonication extraction. The highest enrichment yield was obtained with the Diaion^TM^ HP20LX resin (SI_1_-Di_1_, 50.62%) compared to other fractions under study (Table 2).

**Figure 1:**
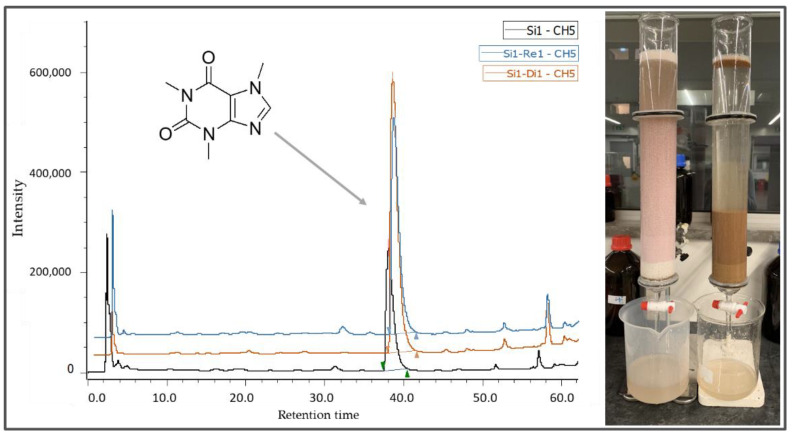
HPLC chromatogram of caffeine before and after enrichment.

The chromatograms obtained from the HPLC analysis of crude and enriched fractions are depicted in Figure 1. The highest caffeine enrichment was observed in the SI_2_ series (SI_2_DI_1_: 4.16-fold and SI_2_RE_1_: 4.10-fold). However, a significantly higher (*p* < 0.05) caffeine content was achieved in SI_1_DI_1_ (1333.78 mg/L vs. SI_2_DI_1_ (1278.45 mg/L) and SI_2_RE_1_ (1259.21 mg/L)).

In addition, the highest phenolic enrichment was also obtained with the Diaion^TM^ HP20LX (SI_1_-Di_1_: 29.15 mg CAE/L; SI_1_-Di:17.09 mg GAE/L). Total phenolics increased, in relation to the crude extract, almost 2.75- and 3.51-fold for chlorogenic acid and gallic acid equivalents, respectively. Overall, a considerable phenolics enrichment was observed in all samples under study. 

Based on the higher extraction yield obtained in the SI_1_ series (~50%), the higher phytochemicals enrichment yield regarding the DI_1_ series, and the considerable enhancement on caffeine and total phenolics content (Table 2), SI_1_DI_1_ was selected as the best enriched fraction to proceed with the studies (Chart 1 and Chart 2).

The results obtained herein are in accordance with those already published by Fathi et al. [5] who reported plant extract enrichment in phenolic compounds using a Diaion HP20 resin. The EXA-118 adsorbent resin was also successfully employed in the purification of anthocyanins and hydroxycinnamic acids (phenolic backbone) from a citrus by-product [36]. Moreover, the use of adsorbent resins in chromatography columns for the pre-purification of food ingredients, as a source of economic technology, was previously described in the food area [37]. Additionally, Diaion HP20 was also previously used for the extraction and purification of esculeoside A (steroidal alkaloid glycosides) from tomato [38]. It must be taken into consideration that Relite EXA90 was not reported previously in the extraction and purification of alkaloids from natural sources. Therefore, this study provides relevant information about the specific functionality of Relite EXA90 on the purification of alkaloid-based natural products (caffeine; purine alkaloid) regarding the considerable information obtained for SI_2_DI_1_ and SI_2_RE_1_ (Table 2).

### 3.2. Physicochemical Properties of Nano/Micro-Particles

#### 3.2.1. Product Yield

The product yield was calculated based on the method described in Section 2.6.5 and Equation (1). It must be taken into consideration that the inlet temperatures were set according to the NP used (Section 2.6.5), ranging among 115 and 135 °C in order to enhance the final product yield and decrease moisture content, resulting in improved storage and stabilization of the particles [39]. The product yields ranged from 35.22 to 72.92%, considering all samples (Table 3). Among the four subgroups under study, no considerable differences were observed. In contrast, the product yield varied from the type of NP and the drying conditions, which might be due to the viscosity, volume, and concentration of the initial feed, drying temperature, features, and ratio of the coating layer, according to Tontul and Topuz [40]. The experimental results showed the high product yield from starch and gum Arabic particles, in both forms, single and complex coating layers. Gum Arabic showed a higher product yield. These results are in accordance with those of Ferreira et al. [41], who reported high product yields (≥50%) for 1:1.5 and 1:2 starch/gum Arabic. The authors reported that increasing the ratio of gum Arabic allows a significant increase on the product yield of a complex of starch/gum Arabic. In contrast, a low product yield was observed for pectin. A significant improvement (almost 4–6%) on the product rate was observed in secondary coating layers (I and II) comparatively to NP in the single pectin coating, while, in the case of the pectin/starch complex, the yield decreased in all categories compared with the single coating (Table 3). It could be concluded that the impact of these factors will depend on the lipid layer, in this case, especially in the nano-phytosome incorporated by cholesterol, to improve the stability of lipid particles in solution media [9], provide the adhesion between the lipid layer and NP, and avoid mass loss during drying [16]. Moreover, the complex of pectin/ starch provides surface coverage of the particles and reduces adhesion during spray drying [42]. These two factors lead to a slight improvement from the NP coating to NP-Secondary coatings I and II, and, resulting in a considerable product yield on PH/CHL-SIDI-PE/STC.

However, the product yield showed slight differences, when comparing the NP first coating and NP-secondary coating I and II. A slight increase in the production yield of some of the NP-secondary coatings II (2–15%) occurred, when compared to the single coating and NP-secondary coating I. Furthermore, no practical differences were observed for NP complexes on coating layers in all samples under study. In general, NP-secondary coating II presented slightly higher product yields compared to the other samples under study.

Meanwhile, a certain powder loss occurred in all samples due to the adhesion of particles to the drying cyclone. This phenomenon occurred due to the low glass transition temperature of the initial ingredient (phytoconstituent) at high drying temperature. Therefore, the drying temperature causes surface deformation of the particles to a viscoelastic rubbery state, causing their adhesion to the drying chamber. Although the addition of NP (with a high glass transition temperature) considerably improves this phenomenon, to decrease the mass loss during the process, an optimization of NP concentration is required, as well as adding secondary coating layers (I and II). The referred situation can justify the low product yield values obtained for some variables. However, some mass loss during the drying process is expected and could not be eliminated [37,39].

#### 3.2.2. Encapsulation Efficiency and Loading Capacity

The encapsulation efficacy (EE%) and loading capacity (LC%) of all formulated samples in powder are presented in Table 3. The highest to lowest average EE% was observed as follows: NP first coating (94.46 ± 0.20–97.15 ± 2.96%) > NP-secondary coating I (92.57 ± 1.57–96.62 ± 0.79%) > NP-secondary coating II (91.22 ± 3.59–95.11 ± 0.17%). The highest EE% was observed for SIDI-STC, and the lowest one was presented by PH/CHL-SIDI-STC (Table 3). The LC followed a similar behavior to the one described for EE%. The highest LC was observed in NP coatings, and the lowest one in NP-secondary coating II (Table 3). In general, the results demonstrated a slight decrease in EE% (although not statistically significant) and a significant decrease in LC% (*p* < 0.05) from the first layers to secondary layers, which may be due to the low encapsulation efficiency of nano-phytosomes and nano-phytosomes incorporated by cholesterol (NP-secondary coatings I and II), as reported by Huanga et al. [43]. These authors verified EE% of 35.88% for phytosome structures. However, NPs in the complex form did not significantly influence EE% nor LC% within each group of samples (Table 3).

As shown in this work, EE% is strongly affected by the initial materials used to manufacture the particles, where trial-and-error is usually required to select the best wall materials, as reported by Gharsallaoui et al. [44]. The slight differences found in EE% (not statistically different, *p* > 0.05) may be due to the first coating layer (lipid coating) and the drying and encapsulation conditions, which confirm the results obtained by Roccia et al. [45], which reported an EE% decrease upon increasing the drying temperature. Therefore, it could be concluded that the drying conditions and the lipid carriers might affect the EE%, but not the type of NP used.

#### 3.2.3. Fourier-Transform Infrared Spectroscopy Analyses (FTIR)

**Figure 2:**
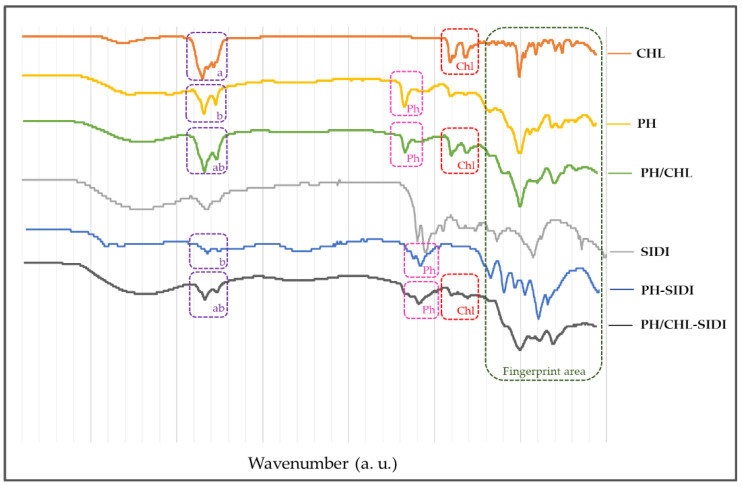
Specific interactions among phosphatidylcholine, cholesterol, and SIDI at PH/CHL, PH-SIDI, and PH/CHL-SIDI by FTIR spectra.

The FTIR spectra was recorded in the scanning range from 400 to 4000 cm^−1^, confirming the interactions among SIDI, lecithin (phosphatidylcholine), and cholesterol in the nano-phytosomes and nano-phytosomes incorporated by cholesterol. The interactions among lipid substrates and SIDI via suppressing a functional group at PH, PH/CHL, PH-SIDI, and PH/CHL-SIDI compared to SIDI, phosphatidylcholine, and cholesterol was proven (Figure 2).

Furthermore, a significant change in the fingerprint area (marked in Figure 2, 400–1500 cm^−1^) and critical substitution areas clearly show the interaction between the lipid bilayer and SIDI. Taking into consideration Figure 2, peaks (a) and (b) show, at the specific critical substitution areas, differences in PH-SIDI compared to PH/CHL-SIDI, confirming the lack of a functional group of cholesterol at PH-SIDI, and the existence of phosphatidylcholine on the related area (2924 cm^−1^). The cholesterol and phosphatidylcholine functional groups at 2924 cm^−1^ in PH/CHL, PH-SIDI, and PH/CHL-SIDI confirmed the interaction of phosphatidylcholine in all related structures.

The following results are in line with those of Hou et al. [46] that confirmed the physicochemical interaction between mitomycin C-soybean phosphatidylcholine by FTIR, paying attention to significant differences between the pure compound and phytosome complex [47]. Moreover, a significant suppression of the sharp endothermic nature of the functional groups of the spectra of curcumin and phospholipid was reported [46], which is in line with the verification in the current FTIR spectra.

#### 3.2.4. Nano/Micro Particle Size Distribution 

The particle size was determined via the effective particles diameter, number, and volume of internal layers. In all samples, the particles were heterogeneous, at nanoscale (≤800 nm). For particle size evaluation, the initial lipid coatings regarding nano-phytosomes (PH-SIDI) and nano-phytosomes incorporated by cholesterol (PH/CHL-SIDI), initial NP coating (SIDI-NPs), and NP-secondary coating (PH-SIDI-NPs and PH/CHL-SIDI-NPs) were considered (Table 4). Equipment from Brookhaven Instruments (Holtsville, NY, USA) evaluated the particle size (internal layers).

##### Initial Lipid Coating

The distribution of particle size on lipid layers was evaluated after 20 min of sonication for both nano-phytosomes (PH-SIDI) and cholesterol-incorporated nano-phytosomes (PH/CHL-SIDI), immediately after the hydration to avoid agglomeration. PH-SIDI and PH/CHL-SIDI had less than 120 nm, with nanoparticles in both volume and number (Table 4). The obtained results are in line with those of Minaei et al. [48], in the case of PH-SIDI, and in agreement to Nazari et al. [49], in the case of PH/CHL-SIDI. The nanoparticles size increased in the presence of cholesterol (PH/CHL-SIDI). Therefore, it was demonstrated that incorporating cholesterol in the lipid bilayer, between phospholipids, increases the particles size, but also improves their stability as reported by Rasaie et al. [9].

##### Initial NP Coating

To crush the NP external coating layer and determine the particle size of the internal layers, a small amount of powder previously dispersed on ethanol 99% was subjected to 7 min of sonication (Table 4). The particles size of the NP external coating is discussed in Section 3.2.5. The particles size of the internal layers was in the range of 187.1–786.8 nm in number and 226.1–808.7 nm in volume. The pectin-based coating produced the biggest particles and the STC and PE/STC complex produced the smallest. Similarities were observed in the distribution of volume and number. Therefore, the particles followed a harmonious distribution at internal layers regarding the initial NP coating, and followed structural arrangements Type A-I and A-II. The probable molecular arrangement of NP coatings were previously discussed [16].

The size-reducing properties of starch in the PE/STC complex is proven in this study and it is in line with the results reported by Fathi et al. [16]. This behavior may be due to the initial features of some carbohydrates such as starch that affect steric hindrance mechanisms on the particles’ surface when placed among the NP molecules. Therefore, this feature could limit the particle size. The impact of starch on gum Arabic was also evaluated. Nevertheless, the observed results with the GA/STC complex showed different outcomes compared to PE/STC. These results are in line with those of Chanamai et al. [50] that reported particles size of the GA/STC complex around 700 nm in emulsion form.

##### NP-Secondary Coating

The evaluation of PH-SIDI-NPs and PH/CHL-SIDI-NPs was described in Section 3.2.4. *Initial Lipid Coating* The particles size was moderately larger compared to the initial NP coatings. The biggest particles were obtained with maltodextrin and the smallest ones with starch in single form. The biggest and smallest complex particles were from GA/STC and PE/STC, respectively. Distributions in volume and number were also similar (as in Section 3.2.4. *Initial NP Coating*). Considering the PH-SIDI (70.8 nm) and PH/CHL-SIDI (114.1 nm) particle size in the secondary coating layer and considering the formation of the coating layer explained by Fathi et al. [12], NP-secondary coating I and II probably follow A-III and A-IV and A-V and A-VI types. In the way, the small differences between volume and number confirm the structure type A-III and A-IV followed by the majority of particles [16]. Moreover, the size-reducing properties of starch in the PE/STC complex was verified in both series as well (Table 4) [51].

The particle size distribution after four months of preparation was recorded to evaluate their stability against moisture in storage conditions (−4 °C, 95% moisture). A slight increase in particles size in all samples under study was obtained. However, the particles were still in an acceptable size range, and no relevant instability was observed.

#### 3.2.5. Surface Morphology of External Layers

**Figure 3:**
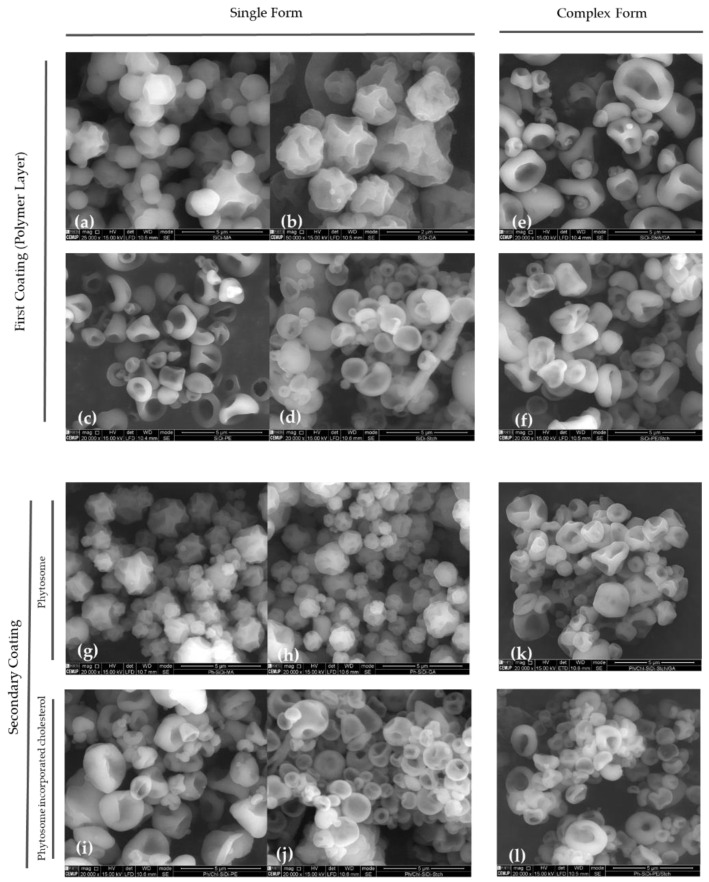
Surface morphology of the final products regarding first and secondary coatings. First coating: (**a**) Maltodextrin; (**b**) gum Arabic; (**c**) pectin; (**d**) starch; (**e**) starch/gum Arabic; (**f**) pectin/starch. Secondary coating: (**g**) Maltodextrin; (**h**) gum Arabic; (**i**) pectin; (**j**) starch; (**k**) starch/ gum Arabic; (**l**) pectin/ starch.

Scanning electron microscopy (SEM) was used to evaluate the surface morphology and determine the particles size of the external layer for all particles under study. The SEM images cannot provide any information regarding internal layers (Figure 3). The surface morphology was quite different regarding each NP in the single form, and complex forms had an intermediate morphology (mixture of both NPs). In summary, the surface morphology shows spherical shrinkage with regular and irregular concavities related to the differences in the NPs shapes. Regular concavities on the particles’ surface were observed in gum Arabic and maltodextrin. An irregular shrinkage, albeit uniform, was observed in starch and pectin particle surfaces in the single matrix (Figure 3). The obtained results concerning maltodextrin were in line with those reported by Papoutsis et al. [52] with slight differences. The authors reported a spherical uniform surface with low concavities in the case of maltodextrin, and the results of this work showed more deep concavities while still spherical and uniform. These differences may be due to differences in the drying temperatures that directly affect the glass transition temperature of NP, resulting in surface deformation. This happened for all samples under study [37,39]. In the case of starch particles, a spherical surface, uniform and regular with some concavities, is in agreement with the uniform irregular surface with a moderate rate of concavities reported by Gangurde et al. [53]. The surface morphology of complex forms followed an intermediate route with a decreasing rate of concavities. The results reported in this study concerning the surface morphology of starch and pectin particles are in accordance with those of Cortes et al. [54].

In terms of the external layer particle size, although no remarkable differences were observed in the particles under study, it ranged 1–5 µm for all samples. These values (the size of external layers) were directly affected by drying conditions, such as inlet and outlet temperatures, the spraying nozzle, and the aspiration rate [40]. Therefore, the similarity between the particle sizes of external coating layers is justified.

#### 3.2.6. Surface Charge of Internal and External Layers

The surface charge (zeta potential) of all particles (nano-phytosome, nano-phytosome incorporated by cholesterol, NP coatings, and NP-Secondary coatings) were evaluated regarding the internal and external layers. A surface charge lower than −30 mV (anionic surface charge) is desirable in terms of stability at aqueous media, compelling repulsion between particles and avoiding agglomeration. On the other hand, the surface charge of about ±10 mV produces neutral particles, favoring the agglomeration rate [55,56]. It must be taken into consideration that cationic particles have more toxicity to the cell wall membrane [55], but by contracting, they could interact better with the cell membrane [57]. The lipid bilayer improves the permeability due to its similarity to the cell membrane [58]. Therefore, the anionic lipid-based particles play a vital role in food and pharmaceutical final products, resulting in improved permeability of active ingredients/drugs and avoiding the probable toxicity related to the cationic particles [24]. Keeping this information in mind, all particles presented a desirable anionic charge, ranging from −24.43 to −61.76 in the case of internal layers (Table 4) and from −15.25 to −58.17 in the case of external layers (Table 4). It is to be noted that the surface negative charge of internal layers was moderately higher than the external layers, in the same conditions. This feature can contribute to the stability of particles, avoiding a high agglomeration rate. The lipid layers surface charge ranged between −84.22 ± 6.58 and −75.06 ± 5.86 mV for the phytosome and the cholesterol-incorporated phytosome, respectively. The negative surface charge of lipid layers, in the case of phosphatidylcholine, was already reported by Hindarto et al. [59], and in the case of phosphatidylcholine bilayer incorporated by cholesterol, by Tang et al. [57].

Considering the values presented in Table 4, the zeta potentials decreased significantly from the lipid layer to NP coatings. In general, a slight increase from the NP coating to NP-Secondary coatings I and II was verified. In terms of performance, the zeta potential of gum Arabic and starch considerably decrease in both complex forms (GA/STC, and PE/STC) (Table 4). However, the pectin zeta potential increases in these forms (PE/STC). The mentioned results show the positive impact of starch on stabilizing the PE/STC complex in solution. The results reported are in line with Fathi et al. [16]. Indeed, it could be concluded that the size-reducing properties of starch could improve the zeta potential, increasing colloidal stability.

#### 3.2.7. In Vitro Drug Release Studies

The in vitro drug release study (dissolution assay) was performed at pH 2.1 and 7.8, corresponding to gastrointestinal media. The profiles obtained by the dissolution assays were evaluated regarding kinetic mathematical models and stabilization of the produced particles in an in vitro environment. Table 5 lists the results obtained at pH 2.1 and Table 6 at pH 7.8. Zero-order (ZO), first-order (FO), Higuchi (HG), Hixson–Crowell (HC), and Korsmeyer–Peppas (KrP) were the theoretical mathematical models used to evaluate the capacity for the prediction of release profiles. The criteria for selecting the most appropriate model were based on the adjusted correlation coefficient in which R^2^_Adj_ ≥ 0.800 was considered as an acceptable fit indicator of the model, and the best-fitting model was the one with the R^2^_Adj_ closest to 1 [32,33,34].

Zero-order kinetics were applied to determine if the caffeine release from the particles followed a Fickian or non-Fickian mechanism. A Fickian mechanism occurs when the bioactive/drug release happens at a constant and linear rate, during a specific time, and independently of the concentration at any time [24]. The zero-order kinetics model supports a sustained-release mechanism, that is, the active agent is released at a constant rate during a period of time. This assures that the active agent is continuously released at a constant pharmacological concentration, but on the other hand, it implies a frequent repetitive dosing, considering the limited amount of the active agent per formulation, which rapidly causes the drug concentration to be outside of the therapeutic range [25]. In relation to the tested formulations at pH 2.1, and according to the R^2^_Adj_ value, it was concluded that the zero-order kinetic model did not fit well to most of the release results, indicating an overall non-Fickian release mechanism with the exception of five formulae: SIDI-MA (R^2^_Adj_: 0.927), PH/CHL-SIDI-MA (R^2^_Adj_: 0.954), PH-SIDI-PE (R^2^_Adj_: 0.964), SIDI-STC (R^2^_Adj_: 0.824), PH/CHL-SIDI-STC (R^2^_Adj_: 0.975). In contrast, regarding pH 7.8, most of the drug release assays of the particles fitted well to the zero-order kinetic model (indicating a Fickian release mechanism), except for the formulations PH/CHL-SIDI-MA (R^2^_Adj_: 0.642), SIDI-GA (R^2^_Adj_: 0.451), PH-SIDI-GA/STC (R^2^_Adj_: 0.697), and PH/CHL-SIDI-GA/STC (R^2^_Adj_: 0.489). For zero-order kinetics models, the release mechanism relies on the fact that the amount of the drug dissolved only depends on time. Thus, the synthetized formulations that were revealed to be compatible with a zero-order kinetic model allow the drug to be dissolved at a constant rate independently of the active agent concentration.

The first-order kinetic model is normally used to mathematically describe the release, absorption, and dissolution phenomena of a drug from a solid particle in a liquid media, thus being dependent on the surface area of the solid in contact with the solution medium [29]. In this process, the drug release rate is concentration dependent [15], resulting in a super slow release profile since the concentration of the drug diminishes with the release process, which in turn slows down due to the lower drug concentration inside the particles [33,60]. By analyzing the calculated statistical parameters, in Table 5 and Table 6, derived from the application of the first-order model to the dissolution assays’ results, it was concluded that the first kinetic model fitted well to the release data associated with pH 2.1 (R^2^_Adj_ ≥ 0.877) and associated with pH 7.8 (R^2^_Adj_ ≥ 0.797) except in the particles SIDI-MA (R^2^_Adj_: 0.470) and PH-SIDI-PE (R^2^_Adj_: 0.569). Therefore, the release of the formulated particles conceptualizes a constant compound release over specific times (super slow release) regarding a good fit of the first-order kinetic model.

The Higuchi model is based on a premier mathematical kinetic equation that describes drug release. Even though this model presented an overall good fit to the in vitro release results, some reservations about its suitability were still assumed. As general knowledge, the Higuchi models are sustained by numerous assumptions such as a scarce or non-existing swelling rate and the erosion of the coating matrix, with the drug release only being based on a Fickian diffusion for water-soluble and low-soluble drugs in the polymer matrix. The root-based Higuchi equation was used when the polymer matrices were swellable and biodegradable, giving rise to a *pseudo-steady-state* as described by Higuchi [33,61]. Most of the calculated results for the Higuchi model (Table 5 and Table 6) revealed a good fit at both the tested pH (R^2^_Adj_ ≥ 0.800), indicating that the release of caffeine from most of the produced particles was dependent on the erosion of the coating matrix. Moreover, it was proved that the produced particles were swellable in both tested pH values, especially at pH 7.8 with the exception of SIDI-GA (R^2^_Adj_: 0.699) and PH/CHL-SIDI-GA/STC (R^2^_Adj_: 0.700), (Table 6).

The Hixson–Crowell model tries to mathematically justify the dissolution rate of uniform particles in a powder by describing the release profile based on the cube root of cumulative concentration of drugs released at time “t”. This equation was adjusted to numerous pharmaceutical products, namely tablets and drug particles [33,60]. Furthermore, this model assumes that the drug’s release velocity is limited by its dissolution rate and not by its diffusion across the polymeric matrix. The Hixson–Crowell model justifies a drug release based on constant sustainability of the geometrical form of particles over time. In other words, the dissolution rate should be constant if the surface morphology of particles (whether cubic or spherical) is also constant. Hence, the samples that fit well with the Hixson–Crowell model have a constant geometrical based dissolution profile [29]. The R^2^_Adj_ value of the Hixson–Crowell model fits better to the result obtained from pH 7.8, except PH/CHL-SIDI-GA (R^2^_Adj_: 0.791) and PH-SIDI-PE (R^2^_Adj_: 0.779), compared to results obtained from pH 2.1 in which particles PH-SIDI-MA (R^2^_Adj_: 0.670), SIDI-PE (R^2^_Adj_: 0.755), SIDI-GA/STC (R^2^_Adj_: 0.687), PH/CHL-SIDI-GA/STC (R^2^_Adj_: 0.670), SIDI-PE/STC (R^2^_Adj_: 0.557) showed the lowest fitting correlation determination. Therefore, the R^2^_Adj_ obtained by fitting the Hixson–Crowell model to the dissolution results indicated that the caffeine release rate occurred by a dissolution process and not by the diffusion on the surface of particles. The prevalence of the dissolution over diffusion probably occurs because when the solution is agitated deeply it prevents the accumulation of particles at the bottom of the solution, resulting in a slow diffusion rate [33]. This phenomenon justified the slow-release time (stabilization time) obtained from most of particles.

The Korsmeyer–Peppas model is most often used to justify the release profile of substances from polymeric matrixes and additionally allow one to classify the release mechanism based on the interpretation of its release exponent (“*n*” in Equation (6)). Korsmeyer–Peppas is a semi-empirical power law equation assuming the particles geometry spherical (6) [15], and when the n value corresponds to 0.43, the release is due to pure Fickian diffusion (Case I) (resulting in low polymer degradation); when n values reach 0.85 then the system release is a Case II transport following a zero-order kinetic, which is dependent on the swelling and relaxation of the polymer chains; when n values are between 0.43 and 0.85, the transport is anomalous, meaning that the mechanism of release involves both Fickian diffusion and Case II transport [29,31,62].

Considering the experimental results in the present work, the Korsmeyer–Peppas model was revealed to have a satisfactory fitting based on R^2^_Adj_ at pH 7.8 (R^2^_Adj_ ≥ 0.819), except SIDI-MA (R^2^_Adj_: 0.477), and moderate satisfaction at pH 2.1 regarding that R^2^_Adj_ ≥ 0.690. The obtained results for this model (Table 5 and Table 6) might be due to the non-linear release data (microcapsules/particles or microspheres), which was the main aim of the application of Korsmeyer–Peppas kinetic models [60].

By observing the calculated release factors, “*n*” values of the Korsmeyer–Peppas model (Table 5 and Table 6), significant differences based on transport mechanisms were observed for all the synthetized carrier-particles under characterization, and additionally, these differences could not be categorized accordingly with the groups of the coating walls exploited in this study. Nevertheless, the drug releases could be divided in just two routes: Case-I drug transport and anomalous drug transport for both pH, with the exception of PH/CHL-SIDI-MA at pH 2.1, which followed super case-II drug transport (*n* = 0.932). When *n* < 0.43, the drug release follows case-I drug transport, justified by the low degradation of the polymers matrix, and as the n value increases, it indicates that the degradation of polymers also increases, giving rise to anomalous super case-II drug transport [15]. A low rate of polymer degradation results in a slow release profile. Therefore, the slow release of most of the particles was justified [15]. However, overall, and considering the magnitude of the tabulated data (Table 5 and Table 6), it was not possible to elaborate profiles of calculated release factors and relate these to the different groups of coating polymers exploited in the study. This way, the specific classification of the particle’s dissolution assay at both pH levels could not be determined. This fact may be related to the diversity of the chemical composition of the NP used for producing the polymer coating walls.

In vitro drug release in terms of stabilization time was observed to be discordant in all samples under study with a slow-release time except in some particles (explained below in the same section). The super slow release may be justified by the concentration of the NP (1:10 and 1:15), which can make the drug’s diffusion through a particle coating difficult (Table 5 and Table 6). Our result is in accordance with the work of Paarakh et al. [33] who reported that the concentration of NP influences the media viscosity and, consequently, the drug’s diffusion, significantly increasing the stabilization time. Furthermore, regarding pH 2.1, no significant differences were observed between the NP coating and NP-Secondary coating II, contrary to almost all particles of the NP-Secondary coating I (phytosome-based) in which the stabilization times were enhanced at current pH (Table 5 and Table 6). Regarding pH 7.8, the stabilization release time varied between (216.1 and 1080.1 min) except for the gum Arabic coating in which the release time was enhanced from the NP coating to NP-Secondary coating II at both pH (ascending order; increase in release stabilization time by adding the coating layer resulting in the improvement of the particle stability and increase in the release time), which can be justified by findings in the work of Paarakh et al. [33] regarding the polymer concentration (explained in beginning of this paragraph). However, in contrast, maltodextrin stabilization times were observed in a descending order in which the release time decreased from the first coating layer (NP-Coating) to NP-Secondary coating II at pH 7.8, as seen in Table 6.

**Figure 4:**
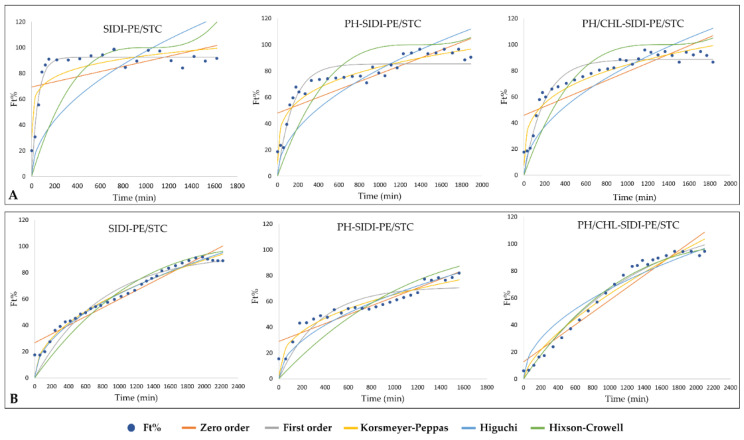
In vitro release assays curves of PE/STC series at two different pH (**A**: pH 2.1; **B**: pH 7.6). The other curves are presented in the Supplementary Materials.

Considering there are numerous kinetic models already established to predict a great variety of release behaviors of different types of micro/nanoparticles, in the present work, different mathematical kinetic models were used to assess their adjustment to the drug release profiles from the several silverskin-based formulations produced [31]. The Korsmeyer–Peppas provided a better understanding about the release behavior of the generally developed carrier’ particles [31,63]. The Korsmeyer–Peppas and first-order kinetic models were able to justify the slow release of most of the as-synthetized particles [15]. In vitro release assays curves of PE/STC series as an example is shown in Figure 4. The other curves are presented in the Supplementary Materials.

#### 3.2.8. In Silico Investigation Based on Molecular Docking

**Figure 5:**
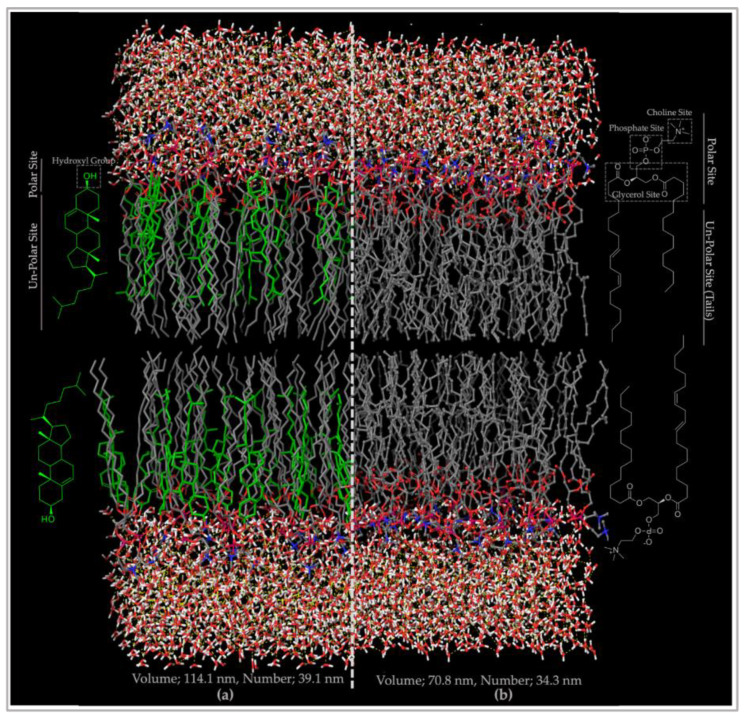
3D model of lipid bilayers. (**a**) Bilayer containing phosphatidylcholine and cholesterol; (**b**) simulated model for bilayer designed with phosphatidylcholine. The polar site of the bilayer is fitted to the outer layer and covered with hundreds of water molecules (the white and red site on top and bottom of bilayer). Differences on bilayer diameter between structure (**a**,**b**) are discussed in Section 3.2.4. *Initial Lipid Coating* and Table 4).

**Figure 6:**
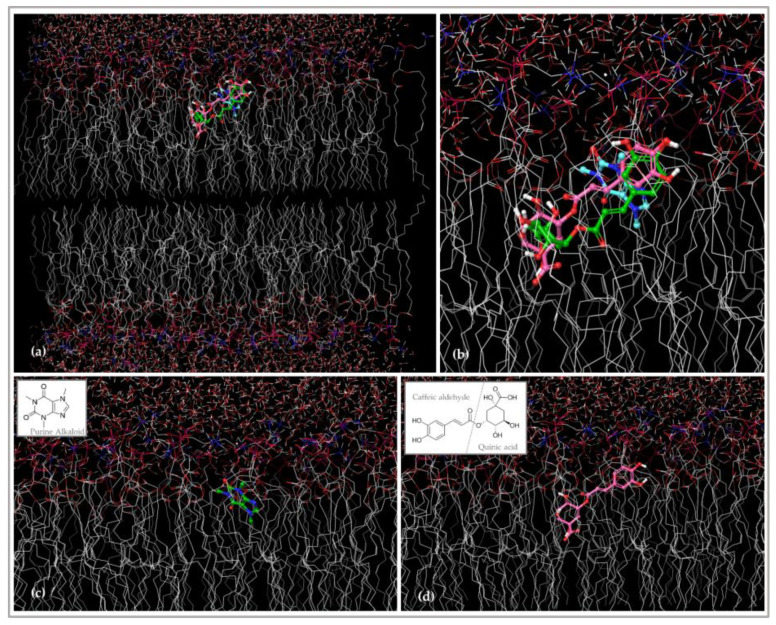
Simulated 3D model of grid of nano-phytosome bilayer docked with main phytochemicals of SI (caffeine and 5-caffeoylquinic acid). In the model, for better visualization, only polar hydrogens are represented. The lipid bilayer appears spherical (see also Figure 4). (**a**,**b**) show an overlap of the compound’s interaction with the lipid bilayer. (**c**) Caffeine (purine alkaloid); adjusted near a polar size with a minimum rate of hydrogen binding. (**d**) 5-Caffeoylquinic acid; the majority of hydrogen binding occurs due to caffeic aldehyde moiety.

**Figure 7:**
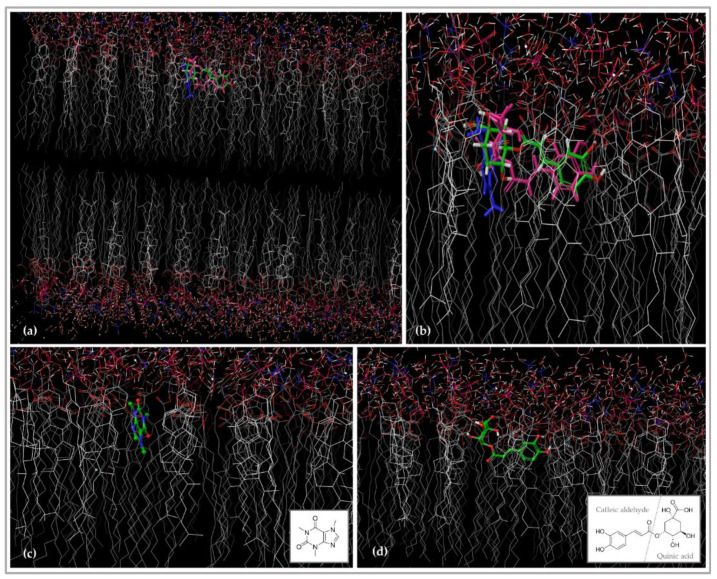
Simulated 3D model of the cholesterol-incorporated nano-phytosome bilayer docked with main phytochemicals of SI (caffeine and 5-caffeoylquinic acid). The lipid bilayer appears spherical (see also Figure 4). (**a**,**b**) show an overlap of the compound’s interaction with the lipid bilayer. (**c**) Caffeine (purine alkaloid); adjusted near a polar size with minimum rate of hydrogen binding. (**d**) 5-Caffeoylquinic acid; the majority of hydrogen binding occurred due to both caffeic aldehyde and quinic acid moieties.

In silico investigation of molecular arrangement was based on caffeine (the main alkaloid structure) and 5-caffeoylquinic acid (also known as chlorogenic acid), which is the main phenolic of SI (Figure 5 and Table 7). The molecular docking confirmed a good interaction between the phytochemicals and the phospholipid bilayer. The molecular arrangement of phospholipid in the nano-phytosome bilayer and cholesterol-incorporated nano-phytosome was designed using the MemGen webserver. The phytoconstituents were docked on both lipid bilayers and evaluated based on a 3D model (Figure 4, Figure 5, Figure 6 and Figure 7), docking score, and grid energy (Table 7), taking into account that a low docking score justifies a high number of hydrogen binding and stronger interactions [64].

The data elicited by molecular docking showed lower and higher docking scores for 5-caffeoylquinic acid and caffeine, respectively, in both lipid bilayers (nano-phytosome and nano-phytosome incorporated by cholesterol) (Table 7). Note that lower docking scores denote higher hydrogen binding. 5-Caffeoylquinic acid exhibited a lower absolute docking score in both structures, although was better suited for the phytosome bilayer (Table 7 and Figure 5 and Figure 6). It was shown that the phytoconstituents were positioned within the lipid bilayer, exhibiting high hydrogen binding between the polar site of the lipid bilayer and the hydroxyl groups of 5-caffeoylquinic acid. Pruchnik et al. [65] also confirmed the interaction between chlorogenic acid and biological membranes, as verified in this work. The referenced authors evaluated the chlorogenic acid permeation mechanism by differential scanning calorimetry and fluorescence spectroscopy, showing the interaction between chlorogenic acid and the polar site of the biological membrane. Therefore, the lower docking score obtained for 5-caffeoylquinic acid (Table 7) is justified.

The interaction between nitrogen and the choline site of phospholipids was confirmed previously by molecular dynamics simulation (MD) regarding the peroxynitrous acid (ONOOH) and phospholipid bilayers [66]. Therefore, the higher docking score suggests the weaker hydrogen binding of caffeine (Table 7). This behavior may be due to its small size, not allowing a good incorporation in the lipid bilayer. However, based on the 3D model (Figure 5 and Figure 6), caffeine was adjusted on the polar site of phosphatidylcholine, just like the chlorogenic acid, but with a low docking score. Phenolics arrangement within a lipid bilayer was reported, recently, for the first time [16]. Based on information reported by Pruchnik et al. [65] and Cordeiro et al. [66] (already discussed in the current section) and the results obtained in this study regarding the in silico molecular arrangement, the interaction between active phytoconstituents/drugs with biological cell membranes could be properly predicted by molecular docking and molecular dynamic simulations.

## 4. Conclusions

The objective of this work was to obtain the highest enrichment of a coffee roasting by-product (SI) extract, followed by its incorporation in nanoparticles. Nano-phytosomes were formulated with the enriched fraction to improve the phytochemicals’ permeation through the cell membrane. The nano-phytosome surface was modified using cholesterol to improve the permeation rate and stability during the formulation process. The structural arrangements were evaluated by in silico molecular docking. To enhance the stability of the formulated structures and natural phytoconstituents, the first lipid-based layer was also coated with NPs. 

The high yield of SI enrichment and the enrichment rate associated with caffeine and total phenolics as main constituents was obtained in the fraction SI_1_DI_1_ and, because of that, it was selected to load the micro/nanoparticles.

Different formulation techniques were used, and the nanoparticles and nano-sized colloids were evaluated regarding physicochemical properties and in vitro drug release. 

The experimental data revealed different formulation groups and significant similarities within related subgroups. In conclusion, higher and lower product yields were observed at ≥70.00% (SIDI-STC and PH-SIDI-GA) and ≤40.00% (SIDI-PE/STC and PH-SIDI-PE/STC), respectively. No significant differences (*p* > 0.05) were found between the EE% of the different groups of particles, while significantly lower (*p* < 0.05) LC% values were observed in NP-secondary coating II (PH/CHL-SIDI-NPs).

Both lipid-based layer structures were confirmed as nanoparticles, and the external layer (NP-coating) varied between 1 and 5 µm for all samples under study. Concerning the inner layer size, it formed various subgroups and varied mainly between 226.1 and 808.7 nm in volume, and from 187 to 786.8 nm, in number.

All formulated particles exhibited anionic surface charges and high stability in aqueous media. FTIR and molecular docking confirmed the interaction of the layers with the purine alkaloid (caffeine) and the phenolic compound (5-caffeoylquinic acid). The best docking score was recorded for 5-caffeoylquinic acid, which exhibited stronger hydrogen bonding, resulting in a better/stronger interaction. 

The in vitro release assays conceptualize the dissolution rate of a specific drug encapsulated in the formulated particles. Considering that the natural polymers’ layers used in this study have good solubility at neutral pH, a higher number of samples with a good fitting of drug release models at pH 7.8 was confirmed. The obtained results allow us to hypothesize that the drug release profile from the particles was pH dependent, and that the coating layers in this work allowed a sustained and controlled drug release at pH 7.8.

Moreover, the majority of the particles followed a super slow release time confirmed by all kinetic models under study following both erosion and diffusion release due to the type of coating layers. Korsmeyer–Peppas, First-order, and Higuchi fitted well to the experimental data regarding the R^2^_adj_ parameter. Furthermore, the majority of formulated particles followed the case-I and anomalous transport mechanism.

To conclude, independently of the type of the coating layer used (first or secondary), the final products can have high efficiency at lower dosages, making them suitable for oral delivery. Based on their enriched content in caffeine and phenolic compounds (antioxidants), they have potential to be used, for instance, in dietary supplements to improve mental and physical performance, competing with other products already existing in the market.

## Figures and Tables

**Table 1:** Encapsulation design: Ratio between ingredients of each formula.

Categories			Formula	Ingredient Ratio			
				SIDI	LEC	CHL	NP
Types of first coating layers	NP-based coating	Single wall layer	SIDI-GA	1	0	0	10
			SIDI-MA				
			SIDI-PE				
			SIDI-STC				
		Complex matrix in wall layer	SIDI-GA/STC	1	0	0	5:5
			SIDI-PE/STC				
	Lipid-based coating	Phytosome (lecithin)	PH-SIDI	1	1	0	0
		Cholesterol incorporated phytosome	PH/CHL-SIDI	1	1	0.5	0
Secondary coating layers	NP-secondary coating I (PH were coated with NP)	Single wall layer	PH-SIDI-GA	1	1	0.5	10
			PH-SIDI-MA				
			PH-SIDI-PE				
			PH-SIDI-STC				
		Complex matrix in wall layer	PH-SIDI-GA/STC	1	1	0.5	5:5
			PH-SIDI-PE/STC				
	NP-secondary coating II (PH/CHL were coated with NP)	Single wall layer	PH/CHL-SIDI-GA	1	1	0.5	15
			PH/CHL-SIDI-MA				
			PH/CHL-SIDI-PE				
			PH/CHL-SIDI-STC				
		Complex matrix in wall layer	PH/CHL-SIDI-GA/STC	1	1	0.5	7.5:7.5
			PH/CHL-SIDI-PE/STC				

SIDI, enriched fraction of the silverskin extract (SI_1_) obtained using the Diaion^TM^ HP20LX resin; PH, nano-phytosome; PH/CHL, cholesterol incorporated nano-phytosome; GA, gum Arabic; MA, maltodextrin; PE, pectin; STC, starch; LEC, lecithin (containing 94% phosphatidylcholine); NP, natural polymer.

**Table 2:** Caffeine and total phenolics content of SI crude and enriched extracts.

Sample	Yield (%)	Caffeine	ECC	Total Phenolics			
				CAE	ECA	GAE	EGA
SI_1_	66.58	432.57 ± 1.64 ^e^	-	10.61 ± 0.42 ^d^	-	4.87 ± 0.28 ^d^	-
SI_1_DI_1_	50.62	1333.78 ± 5.51 ^a^	3.08	29.15 ± 1.54 ^a^	2.75	17.09 ± 1.01 ^a^	3.51
SI_1_DI_2_	DC	7.62 ± 0.18 ^g^	-	6.54 ± 0.39 ^e^	-	2.19 ± 0.26 ^e^	-
SI_1_RE_1_	52.87	951.76 ± 3.87 ^d^	2.20	26.27 ± 1.61 ^b^	2.48	15.19 ± 1.06 ^b^	3.12
SI_1_RE_2_	DC	6.73 ± 0.19 ^g^	-	4.27 ± 0.27 ^ef^	-	0.70 ± 0.18 ^ef^	-
SI_2_	36.67	307.34 ± 1.10 ^f^	-	9.30 ± 0.24^d^	-	4.01 ± 0.16 ^d^	-
SI_2_DI_1_	20.00	1278.45 ± 5.28 ^b^	4.16	19.13 ± 0.67 ^c^	2.06	10.48 ± 0.44 ^c^	2.61
SI_2_DI_2_	DC	6.94 ± 0.19 ^g^	-	5.97 ± 1.17 ^ef^	-	1.82 ± 0.77 ^ef^	-
SI_2_RE_1_	18.00	1259.21 ± 5.19 ^c^	4.10	18.23 ± 0.86 ^c^	1.96	9.89 ± 0.57 ^c^	2.47
SI_2_RE_2_	DC	6.74 ± 0.19 ^g^	-	3.80 ± 0.22 ^f^	-	0.38 ± 0.15 ^f^	-

Values of caffeine, CAE, and GAE expressed in mg/L (mean ± standard deviation). Different letters within the same column represent significant differences between samples at *p* < 0.05. ECC, enhance on caffeine content; ECA, enhance on chlorogenic acid content; EGA, enhance on gallic acid content; CAE, chlorogenic acid equivalents; GAE, gallic acid equivalents; DC, discarded fractions containing sugar and chlorophylls.

**Table 3:** Product yield, encapsulation efficiency, and loading capacity of formulated samples in powder.

	Formula	Yield (%)	EE (%)	LC (%)
Lipid-based first coating	PH-SIDI	60.22	-	-
	PH/CHL-SIDI	57.44	-	-
NP-based first coating	SIDI-GA	50.00	95.67 ± 1.08 ^a^	8.70 ± 0.10 ^a^
	SIDI-MA	42.55	96.51 ± 1.10 ^a^	8.77 ± 0.10 ^a^
	SIDI-PE	42.36	95.02 ± 0.02 ^a^	8.64 ± 0.00 ^a^
	SIDI-STC	72.18	97.15 ± 2.96 ^a^	8.83 ± 0.27 ^a^
	SIDI-GA/STC	66.75	94.46 ± 0.20 ^a^	8.59 ± 0.02 ^a^
	SIDI-PE/STC	37.25	95.43 ± 1.60 ^a^	8.68 ± 0.15 ^a^
NP-secondary coating I	PH-SIDI-GA	72.92	94.26 ± 0.24 ^a^	7.85 ± 0.02 ^a^
	PH-SIDI-MA	51.69	94.68 ± 1.24 ^a^	7.89 ± 0.10 ^a^
	PH-SIDI-PE	46.92	96.62 ± 0.79 ^a^	8.05 ± 0.07 ^a^
	PH-SIDI-STC	48.31	92.57 ±1.57 ^a^	7.71 ± 0.13 ^a^
	PH-SIDI-GA/STC	62.67	94.26 ± 1.46 ^a^	7.86 ± 0.12 ^a^
	PH-SIDI-PE/STC	35.22	94.29 ± 1.04 ^a^	7.86 ± 0.09 ^a^
NP-secondary coating II	PH/CHL-SIDI-GA	65.51	92.56 ± 2.63 ^a^	5.29 ± 0.15 ^b^
	PH/CHL-SIDI-MA	53.62	94.64 ± 1.25 ^a^	7.02 ± 2.35 ^ab^
	PH/CHL-SIDI-PE	50.70	95.11 ± 0.17 ^a^	5.43 ± 0.01 ^b^
	PH/CHL-SIDI-STC	54.49	91.22 ±3.59 ^a^	5.21 ± 0.21 ^b^
	PH/CHL-SIDI-GA/STC	61.73	93.61 ± 0.53 ^a^	5.35 ± 0.03 ^b^
	PH/CHL-SIDI-PE/STC	42.38	94.44 ± 0.96 ^a^	5.40 ± 0.05 ^b^

Different letters within the same column represent significant differences between samples at *p* < 0.05. SIDI, enriched fraction of the silverskin extract (SI_1_) obtained using the Diaion^TM^ HP20LX resin; PH, nano-phytosome; PH/CHL, cholesterol incorporated nano-phytosome; GA, gum Arabic; MA, maltodextrin; PE, pectin; STC, starch; NP, natural polymer. Note: EE% and LC% were not recorded for PH-SIDI and PH/CHL-SIDI.

**Table 4:** Particle size (internal layers, nm) and zeta potential (mV) of internal and external layers.

Formula Name	Particle Size (nm)								Zeta Potential (mV) *	
	First Month (Internal Layers)		kcps	PDI	Fourth Month (Internal Layers)		kcps	PDI		
	Volume *	Number			Volume *	Number			Internal Layers	External Layers
PH-SIDI	70.8 ± 0.3	34.3	399.3	0.161	74.1 ± 0.8	36.6	513.3	0.157	−84.22 ± 6.58	
PH/CHL-SIDI	114.1 ± 1.1	39.1	459.6	0.235	179.1 ± 1.0	70.0	536.9	0.206	−75.06 ± 5.86	
SIDI-GA	512.4 ± 62.7	498.6	502.9	0.005	691.3 ± 74.1	672.6	443.3	0.005	−51.02 ± 4.43	−53.54 ± 3.65
SIDI-MA	619.1 ± 147.7	602.3	322.6	0.005	832.3 ± 90.8	602.3	396.9	0.005	−33.83 ± 16.96	−25.42 ± 7.44
SIDI-PE	808.7 ± 102.4	786.8	415.8	0.005	892.3 ± 124.7	868.2	528.2	0.005	−30.07 ± 7.47	−45.28 ± 6.89
SIDI-STC	226.1 ± 28.5	187.1	363.6	0.055	267.3 ± 30.3	260.0	433.2	0.005	−52.79 ± 2.97	−58.17 ± 2.97
SIDI-GA/STC	699.8 ± 98.1	680.0	412.4	0.005	919.8 ± 100.1	894.0	462.3	0.005	−49.22 ± 2.82	−49.22 ± 2.82
SIDI-PE/STC	241.8 ± 26.3	235.2	426.3	0.005	267.7 ± 11.1	260.1	473.7	0.005	−37.69 ± 4.00	−45.41 ± 3.49
PH-SIDI-GA	462.8 ± 71.1	404.2	432.4	0.005	650.0 ± 100.1	632.4	422.4	0.005	−61.76 ± 4.48	−52.25 ± 2.83
PH-SIDI-MA	535.2 ± 194.9	520.7	388.9	0.005	893.3 ± 34.0	869.1	351.2	0.005	−31.49 ± 4.73	−19.31 ± 8.73
PH-SIDI-PE	492.0 ± 52.8	478.7	353.7	0.005	588.0 ± 44.0	572.1	386.3	0.005	−30.66 ± 3.67	−19.08 ± 10.79
PH-SIDI-STC	298.7 ± 28.7	290.6	298.7	0.005	327.0 ± 38.9	318.2	439.7	0.005	−52.88 ± 3.11	−32.61 ± 18.30
PH-SIDI-GA/STC	760.1 ± 132.5	505.1	462.7	0.005	976.8 ± 104.1	950.0	465.0	0.005	−39.41 ± 5.39	−31.80 ± 6.67
PH-SIDI-PE/STC	452.0 ± 86.4	439.8	412.4	0.005	688.5 ± 101.2	669.9	412.4	0.005	−33.01 ± 5.85	−31.28 ± 8.38
PH/CHL-SIDI-GA	429.4 ± 45.6	414.8	388.2	0.005	474.3 ± 49.2	461.4	432.5	0.005	−55.22 ± 1.14	−48.43 ± 5.64
PH/CHL-SIDI-MA	604.5 ± 67.7	588.2	361.1	0.005	691.1 ± 96.4	259.0	450.6	0.215	−24.43 ± 12.00	−26.69 ± 9.68
PH/CHL-SIDI-PE	524.2 ± 52.1	527.5	449.4	0.005	626.4 ± 12.8	609.4	449.4	0.005	−31.61 ± 14.35	−15.25 ± 5.87
PH/CHL-SIDI-STC	259.1 ± 8.8	252.1	523.6	0.005	414.2 ± 19.1	403.0	459.2	0.005	−59.86 ± 4.41	−47.95 ± 5.56
PH/CHL-SIDI-GA/STC	441.1 ± 19.2	429.2	460.1	0.005	526.9 ± 36.2	512.6	460.1	0.005	−51.25 ± 4.25	−45.43 ± 7.81
PH/CHL-SIDI-PE/STC	387.4 ± 35.9	376.9	490.3	0.005	435.9 ± 62.4	424.1	500.9	0.005	−34.69 ± 2.31	−30.68 ± 1.95

* The presented values correspond to the mean ± standard deviation provided by the equipment (Brookhaven Instruments, Holtsville, NY, USA). kcps, average count rate (kilo counts per second) that shows a quality of particle size, in which the kcps range approximately between 300 and 500 (it provides a high precise particle size recording); PDI, polydispersity index that shows the size range index of particles: <0.05, high monodisperse standard in which near to zero shows a perfect uniformed sample; >0.7, very broad particle size distribution in which near to 1.0 shows a super highly polydisperse sample with various particle sizes [15].

**Table 5:** In vitro release assays at pH 2.1.

Variable		MA			GA			PE			STC			GA/STC			PE/STC		
		SIDI	PH	PH/CHL	SIDI	PH	PH/CHL	SIDI	PH	PH/CHL	SIDI	PH	PH/CHL	SIDI	PH	PH/CHL	SIDI	PH	PH/CHL
ST (min)		2670.1	540.1	1620.1	210.1	450.1	630.1	270.1	1688.1	522.1	1880.1	1800.1	1860	340.1	900.1	210.1	520.1	1230.1	1298.1
ZO	F_0_	13.554	55.964	2.3204	31.877	49.773	29.398	57.423	15.517	53.611	49.120	44.165	7.946	71.305	54.364	49.044	69.368	48.045	45.833
	K_0_	0.027	0.013	0.052	0.162	0.072	0.045	0.023	0.044	0.029	0.024	0.032	0.046	0.019	0.0427	0.018	0.020	0.30	0.033
	R^2^	0.938	0.321	0.956	0.679	0.697	0.766	0.469	0.965	0.442	0.829	0.735	0.975	0.285	0.619	0.361	0.2618	0.708	0.699
	R^2^_Adj_	0.927	0.297	0.954	0.639	0.673	0.746	0.453	0.964	0.420	0.824	0.726	0.975	0.254	0.600	0.31	0.2230	0.699	0.689
FO	K_1_	0.0004	0.0072	0.0002	0.006	0.0074	0.0065	0.006	0.001	0.0067	0.004	0.003	0.0003	0.009	0.008	0.012	0.018	0.007	0.0051
	K_max_	135.9	75.82	346.5	106.68	95.485	58.959	87.742	123.62	91.992	85.267	90.691	219.803	93.698	86.740	63.917	92.398	85.443	88.626
	R^2^	0.980	0.908	0.960	0.884	0.945	0.897	0.928	0.951	0.953	0.881	0.916	0.978	0.953	0.891	0.895	0.925	0.868	0.941
	R^2^_Adj_	0.977	0.905	0.959	0.869	0.941	0.889	0.926	0.949	0.951	0.877	0.913	0.977	0.951	0.886	0.888	0.921	0.863	0.939
HG	K_H_	1.686	2.136	2.009	4.890	4.049	2.439	2.625	2.075	2.722	2.0283	2.457	1.933	3.008	3.390	2.515	3.073	2.574	2.629
	R^2^	0.944	0.520	0.907	0.808	0.868	0.874	0.649	0.955	0.648	0.942	0.830	0.933	0.530	0.802	0.556	0.499	0.846	0.858
	R^2^_Adj_	0.944	0.520	0.907	0.808	0.868	0.874	0.649	0.955	0.648	0.942	0.830	0.933	0.530	0.802	0.556	0.499	0.846	0.858
HC	K_HC_	0.0002	0.0006	0.0003	0.002	0.002	0.0005	0.0008	0.0003	0.0008	0.0005	0.0006	0.0003	0.001	0.0013	0.0005	0.001	0.0007	0.0007
	R^2^	0.976	0.670	0.941	0.909	0.960	0.938	0.755	0.946	0.839	0.923	0.880	0.959	0.687	0.842	0.471	0.557	0.848	0.926
	R^2^_Adj_	0.976	0.670	0.941	0.909	0.960	0.838	0.755	0.946	0.839	0.923	0.880	0.959	0.687	0.842	0.471	0.557	0.848	0.926
KrP	K_kp_	0.465	27.871	0.089	7.084	19.556	10.643	24.663	0.929	19.555	15.812	10.120	0.159	33.097	16.898	23.889	40.193	16.236	13.418
	N	0.664	0.139	0.932	0.435	0.244	0.260	0.181	0.613	0.218	0.231	0.301	0.849	0.149	0.249	0.149	0.123	0.236	0.266
	R^2^	0.965	0.700	0.957	0.807	0.892	0.828	0.790	0.965	0.759	0.946	0.890	0.979	0.758	0.830	0.737	0.706	0.894	0.905
	R^2^_Adj_	0.960	0.690	0.955	0.783	0.883	0.814	0.783	0.964	0.750	0.944	0.886	0.978	0.748	0.822	0.721	0.691	0.890	0.902
DTM		ANM	C-I	C-II	ANM	C-I	C-I	C-I	ANM	C-I	C-I	C-I	ANM	C-I	C-I	C-I	C-I	C-I	C-I

SIDI, enriched fraction of SI used in all formulated samples; PH, phytosome; PH/CHL, cholesterol-incorporated phytosome; GA, gum Arabic; MA, maltodextrin; PE, pectin; STC, starch; min; minutes; ZO, zero-order; FO, first-order; KrP, Korsmeyer–peppas; HG, Higuchi; HC, Hixson–Crowell; ST, stabilization time (approximately the time when the release start to be stable and the curve change to be linear); R^2^_Adj_, R^2^_Adjusted_; DTM, drug transport mechanism; C-I, case-I; ANM, anomalous; C-II, case-II transport; SC-II, super case-II.

**Table 6:** In vitro release assays at pH 7.8.

Variable		MA			GA			PE			STC			GA/STC			PE/STC		
		SIDI	PH	PH/CHL	SIDI	PH	PH/CHL	SIDI	PH	PH/CHL	SIDI	PH	PH/CHL	SIDI	PH	PH/CHL	SIDI	PH	PH/CHL
ST (min)		500.1	100.1	50.1	54.1	239.1	1229.1	1634.1	1514.1	1594.1	2160.1	2107.1	2160.1	1080.1	1080.1	1080.1	1900.1	1260	1944.1
ZO	F_0_	76.984	28.249	38.031	66.060	43.196	38.309	39.624	52.759	40.692	14.169	16.400	7.122	27.114	44.0878	56.337	26.737	28.941	12.727
	K_0_	0.031	0.412	0.461	0.250	0.190	0.178	0.036	0.023	0.037	0.034	0.037	0.036	0.059	0.046	0.035	0.033	0.035	0.046
	R^2^	0.827	0.956	0.663	0.475	0.835	0.980	0.910	0.838	0.880	0.961	0.910	0.947	0.913	0.709	0.508	0.952	0.887	0.930
	R^2^_Adj_	0.816	0.952	0.642	0.451	0.826	0.979	0.907	0.834	0.975	0.959	0.907	0.945	0.910	0.697	0.489	0.951	0.882	0.927
FO	K_1_	0.010	0.023	0.028	0.097	0.012	0.0009	0.003	0.030	0.003	0.001	0.001	0.0004	0.003	0.007	0.009	0.001	0.003	0.0008
	K_max_	97.320	77.731	99.845	94.204	97.688	371.43	87.869	77.297	93.081	126.21	120.03	156.62	90.655	86.158	89.574	94.589	71.232	124.290
	R^2^	0.502	0.813	0.962	0.874	0.935	0.982	0.887	0.580	0.940	0.986	0.964	0.982	0.957	0.936	0.965	0.961	0.850	0.984
	R^2^_Adj_	0.470	0.797	0.959	0.869	0.931	0.982	0.882	0.569	0.937	0.986	0.963	0.982	0.956	0.933	0.964	0.960	0.844	0.983
HG	K_H_	4.141	7.076	8.859	10.234	6.069	6.759	2.521	2.475	2.564	1.799	1.922	1.740	2.762	3. 101	3.201	2.022	2.078	2.108
	R^2^	0.865	0.912	0.837	0.699	0.944	0.967	0.989	0.903	0.977	0.963	0.934	0.950	0.977	0.862	0.700	0.978	0.933	0.956
	R^2^_Adj_	0.865	0.912	0.837	0.699	0.944	0.967	0.989	0.903	0.977	0.963	0.934	0.950	0.977	0.862	0.700	0.978	0.933	0.956
HC	K_HC_	0.0017	0.0036	0.0076	0.0093	0.003	0.0016	0.0005	0.0007	0.0006	0.0002	0.0003	0.0002	0.0006	0.001	0.012	0.0003	0.0003	0.0003
	R^2^	0.802	0.919	0.969	0.875	0.971	0.791	0.9562	0.779	0.976	0.986	0.968	0.984	0.958	0.919	0.877	0.982	0.904	0.987
	R^2^_Adj_	0.802	0.919	0.969	0.875	0.971	0.791	0.9562	0.779	0.976	0.986	0.968	0.984	0.958	0.919	0.877	0.982	0.904	0.987
KrP	K_kp_	71.286	14.051	16.452	46.261	15.323	2.331	11.684	30.156	9.538	0.485	0.605	0.211	4.119	14.235	23.560	2.416	5.371	0.613
	N	0.043	0.343	0.367	0.157	0.323	0.657	0.279	0.141	0.312	0.676	0.658	0.781	0.440	0.267	0.198	0.475	0.361	0.671
	R^2^	0.508	0.857	0.868	0.827	0.912	0.985	0.976	0.899	0.964	0.980	0.944	0.966	0.978	0.910	0.809	0.975	0.919	0.964
	R^2^_Adj_	0.477	0.845	0.860	0.819	0.907	0.984	0.975	0.897	0.962	0.979	0.942	0.965	0.977	0.906	0.809	0.974	0.915	0.962
DTM		C-I	C-I	C-I	C-I	C-I	ANM	C-I	C-I	C-I	ANM	ANM	ANM	ANM	C-I	C-I	ANM	C-I	ANM

SIDI, enriched fraction of SI used in all formulated samples; PH, phytosome; PH/CHL, cholesterol-incorporated phytosome; GA, gum Arabic; MA, maltodextrin; PE, pectin; STC, starch; min; minutes; ZO, zero-order; FO, first-order; KrP, Korsmeyer–peppas; HG, Higuchi; HC, Hixson–Crowell; ST, stabilization time (approximately the time when the release start to be stable and the curve change to be linear); R^2^_Adj_, R^2^_Adjusted_; DTM, drug transport mechanism; C-I, case-I; ANM, anomalous; C-II, case-II transport; SC-II, super case-II.

**Table 7:** Binding energy of docked molecule expressed as kcal/mol.

Lipid Structure	Categories	Main Compounds	Docking Score *	Glide Evdw	Glide Energy
Phytosome	Alkaloid	Caffeine	−2.518	−20.202	−21.949
	Phenolic	5-Caffeoylquinic acid	−9.008	−26.590	−39.743
Cholesterol-incorporated phytosome	Alkaloid	Caffeine	−2.347	−17.696	−21.247
	Phenolic	5-Caffeoylquinic acid	−7.120	−25.621	−39.665

* Docking score exhibited the number of hydrogen bindings occurring between structures. Lower docking scores denote a higher hydrogen binding.

## Data Availability

The data presented in this study are available on request from the corresponding author. The data are not publicly available due to privacy restriction.

## Supplementary Materials

The supporting information contain the in vitro release assays curves of all samples can be downloaded at: https://www.mdpi.com/article/10.3390/pharmaceutics14010112/s1, Figure S1: In vitro release assays curves associated to MA series at pH 2.1; Figure S2: In vitro release assays curves associated to MA series at pH 7.8; Figure S3: In vitro release assays curves associated to GA series at pH 2.1; Figure S4: In vitro release assays curves associated to GA series at pH 7.8; Figure S5: In vitro release assays curves associated to STC series at pH 2.1; Figure S6: In vitro release assays curves associated to STC series at pH 7.8; Figure S7: In vitro release assays curves associated to GA/STC series at pH 2.1; Figure S8: In vitro release assays curves associated to GA/STC series at pH 7.8; Figure S9: In vitro release assays curves associated to PE series at pH 2.1; Figure S10: In vitro release assays curves associated to PE series at pH 7.8.

## References

[1] Rashid N., Ashraf I., Ramzan S., Bhat R., Qadri H., Wani K., Dar G., Mehmood M. (2021). Impacts of Food Industrial Wastes on Soil and Its Utilization as Novel Approach for Value Addition. Research Anthology on Food Waste Reduction and Alternative Diets for Food and Nutrition Security.

[2] Comunian T.A., Silva M.P., Souza C.J.F. (2021). The use of food by-products as a novel for functional foods: Their use as ingredients and for the encapsulation process. Trends Food Sci. Technol..

[3] Costa A.S.G., Alves R.C., Vinha A.F., Barreira S.V.P., Nunes M.A., Cunha L.M., Oliveira M.B.P.P. (2014). Optimization of antioxidants extraction from coffee silverskin, a roasting by-product, having in view a sustainable process. Ind. Crop. Prod..

[4] Costa A.S.G., Alves R.C., Vinha A.F., Costa E., Costa C.S.G., Nunes M.A., Almeida A.A., Santos-Silva A., Oliveira M.B.P.P. (2018). Nutritional, chemical and antioxidant/pro-oxidant profiles of silverskin, a coffee roasting by-product. Food Chem..

[5] Fathi F., Ebrahimi S.N. Investigation of physiochemical properties nanophytosome obtained from of polyphenolic enrich fraction of Satureja khuzistanica by freeze-drying. Nashrieh Shimi va Mohandesi Shimi Iran. https://www.nsmsi.ir/article_242399.html?lang=en.

[6] Grgić J., Šelo G., Planinić M., Tišma M., Bucić-Kojić A. (2020). Role of the encapsulation in bioavailability of phenolic compounds. Antioxidants.

[7] Amin T., Bhat S.V. (2012). A review on phytosome technology as a novel approach to improve the bioavailability of nutraceuticals. Int. J. Adv. Res. Technol..

[8] Ozkan G., Kostka T., Esatbeyoglu T., Capanoglu E. (2020). Effects of Lipid-Based Encapsulation on the Bioaccessibility and Bioavailability of Phenolic Compounds. Molecules.

[9] Rasaee S., Ghanbarzadeh S., Mohammadi M., Hamishehkar H. (2014). Nano phytosomes of quercetin: A promising formulation for fortification of food products with antioxidants. Pharm. Sci..

[10] Liu N., Park H.-J. (2009). Chitosan-coated nanoliposome as vitamin E carrier. J. Microencapsul..

[11] Hosseini H., Tajiani Z., Jafari S.M., Galanakis C.M. (2019). Improving the shelf-life of food products by nano/micro-encapsulated ingredients. Food Quality and Shelf Life.

[12] Bessada S.M., Alves R.C., Oliveira M.B.P. (2018). Caffeine-based food supplements and beverages: Trends of consumption for performance purposes and safety concerns. Int. Food Res..

[13] Plapied L., Duhem N., des Rieux A., Préat V. (2011). Fate of polymeric nanocarriers for oral drug delivery. Curr. Opin. Colloid Interface Sci..

[14] Wu W., Wang J., Lin Z., Li X., Li J. (2014). Tumor-Acidity Activated Surface Charge-Conversion of Polymeric Nanocarriers for Enhanced Cell Adhesion and Targeted Drug Release. Macromol. Rapid Commun..

[15] Danaei M., Dehghankhold M., Ataei S., Hasanzadeh Davarani F., Javanmard R., Dokhani A., Khorasani S., Mozafari M. (2018). Impact of particle size and polydispersity index on the clinical applications of lipidic nanocarrier systems. Pharmaceutics.

[16] Fathi F., Ebrahimi S.N., Valadão A.I.G., Andrade N., Costa A.S.G., Silva C., Fathi A., Salehi P., Martel F., Alves R.C. (2021). Exploring *Gunnera tinctoria*: From Nutritional and Anti-Tumoral Properties to Phytosome Development Following Structural Arrangement Based on Molecular Docking. Molecules.

[17] Puga H., Alves R.C., Costa A.S.G., Vinha A.F., Oliveira M.B.P. (2017). Multi-frequency multimode modulated technology as a clean, fast, and sustainable process to recover antioxidants from a coffee by-product. J. Clean. Prod..

[18] Ebrahimi S.N., Gafner F., Dell’Acqua G., Schweikert K., Hamburger M. (2011). Flavone 8-C-Glycosides from *Haberlea rhodopensis* Friv.(Gesneriaceae). Helv. Chim. Acta..

[19] Shehzad O., Jin Ha I., Park Y., Wan Ha Y., Shik Kim Y. (2011). Development of a rapid and convenient method to separate eight ginsenosides from *Panax ginseng* by high-speed counter-current chromatography coupled with evaporative light scattering detection. J. Sep. Sci..

[20] Singh R.P., Gangadharappa H.V., Mruthunjaya K. (2018). Phytosome complexed with chitosan for gingerol delivery in the treatment of respiratory infection: In vitro and in vivo evaluation. Eur. J. Pharm. Sci..

[21] Anwar E., Farhana N. (2018). Formulation and Evaluation of Phytosome-Loaded Maltodextrin-Gum Arabic Microsphere System for Delivery of *Camellia sinensis* Extract. J. Young Pharm..

[22] Tuyen C.K., Nguyen M.H., Roach P.D., Stathopoulos C.E. (2014). Microencapsulation of Gac oil: Optimisation of spray drying conditions using response surface methodology. Powder Technol..

[23] Santana A.A., Oliveira R.A.d., Kurozawa L.E., Park K.J. (2014). Microencapsulation of pequi pulp by spray drying: Use of modified starches as encapsulating agent. Eng. Agricola.

[24] Campos J.C., Ferreira D.C., Lima S., Reis S., Costa P.J. (2019). Swellable polymeric particles for the local delivery of budesonide in oral mucositis. Int. J. Pharm..

[25] Nagarwal R.C., Kumar R., Pandit J.K. (2012). Chitosan coated sodium alginate–chitosan nanoparticles loaded with 5-FU for ocular delivery: In vitro characterization and in vivo study in rabbit eye. Eur. J. Pharm. Sci..

[26] Boidya J., Hasan I., Reza M.S. (2020). Carvedilol Matrix Tablet: Formulation and In Vitro Assessment. Bangladesh Pharm. J..

[27] Arisoy S., Comoglu T. (2020). Kinetic evaluation of L-Dopa loaded WGA-grafted nanoparticles. Med. Sci..

[28] Minisy I.M., Salahuddin N.A., Ayad M.M. (2020). In vitro release study of ketoprofen-loaded chitosan/polyaniline nanofibers. Polym. Bull..

[29] Costa P., Lobo J.M.S. (2001). Modeling and comparison of dissolution profiles. Eur. J. Pharm. Sci..

[30] Costa A.L.d.O., Enéas P.C.R., Miranda T.A., Mingoti S.A., Soares C.D.V., Pianetti G.A. (2013). In vitro dissolution kinetic for mycophenolic acid derivatives tablets. Braz. J. Pharm. Sci..

[31] Permanadewi I., Kumoro A., Wardhani D., Aryanti N. (2019). Modelling of controlled drug release in gastrointestinal tract simulation. J. Phys. Conf. Ser..

[32] Rehman Q., Akash M.S.H., Rasool M.F., Rehman K., Akash M.S.H., Rehman K. (2020). Role of Kinetic Models in Drug Stability. Drug Stability and Chemical Kinetics.

[33] Paarakh M.P., Jose P.A., Setty C., Christoper G.P. (2018). Release kinetics–concepts and applications. Int. J. Pharm. Res. Technol.

[34] Cojocaru V., Ranetti A.E., Hinescu L.G., Ionescu M., Cosmescu C., Poștoarcă A.G., Cinteză L.O. (2015). Formulation and evaluation of in vitro release kinetics of Na3CaDTPA decorporation agent embedded in microemulsion-based gel formulation for topical delivery. Farmacia.

[35] Knight C.J., Hub J.S. (2015). MemGen: A general web server for the setup of lipid membrane simulation systems. Bioinformatics.

[36] Scordino M., Di Mauro A., Passerini A., Maccarone E. (2005). Selective recovery of anthocyanins and hydroxycinnamates from a byproduct of citrus processing. J. Agric. Food Chem..

[37] Mahato N., Sinha M., Sharma K., Koteswararao R., Cho M.H. (2019). Modern extraction and purification techniques for obtaining high purity food-grade bioactive compounds and value-added co-products from citrus wastes. Foods.

[38] Fujiwara Y., Takaki A., Uehara Y., Ikeda T., Okawa M., Yamauchi K., Ono M., Yoshimitsu H., Nohara T. (2004). Tomato steroidal alkaloid glycosides, esculeosides A and B, from ripe fruits. Tetrahedron.

[39] Jovanović A.A., Lević S.M., Pavlovic V.B., Markovic S.B., Pjanovic R.V., Đorđević V.B., Nedović V., Bugarski B.M. (2021). Freeze vs. Spray Drying for Dry Wild Thyme (*Thymus serpyllum* L.) Extract Formulations: The Impact of Gelatin as a Coating Material. Molecules.

[40] Tontul I., Topuz A. (2017). Spray-drying of fruit and vegetable juices: Effect of drying conditions on the product yield and physical properties. Trends Food Sci Technol..

[41] Ferreira N.G., Pereira Martin L.G., Matta Fakhouri F., Oliveira A.d.R. (2018). Microencapsulation of blackberry pulp with arrowroot starch and gum arabic mixture by spray drying. J. Microencapsul..

[42] Wang Y., Xie Y., Xu D., Lin X., Feng Y., Hong Y. (2014). Hydroxypropyl methylcellulose reduces particle adhesion and improves recovery of herbal extracts during spray drying of Chinese herbal medicines. Dry. Technol..

[43] Huang Z., Brennan C.S., Zhao H., Liu J., Guan W., Mohan M.S., Stipkovits L., Zheng H., Kulasiri D. (2020). Fabrication and assessment of milk phospholipid-complexed antioxidant phytosomes with vitamin C and E: A comparison with liposomes. Food Chem..

[44] Gharsallaoui A., Roudaut G., Chambin O., Voilley A., Saurel R. (2007). Applications of spray-drying in microencapsulation of food ingredients: An overview. Food Res. Int..

[45] Roccia P., Martínez M.L., Llabot J.M., Ribotta P.D. (2014). Influence of spray-drying operating conditions on sunflower oil powder qualities. Powder Technol..

[46] Hou Z., Li Y., Huang Y., Zhou C., Lin J., Wang Y., Cui F., Zhou S., Jia M., Ye S. (2013). Phytosomes loaded with mitomycin C-soybean phosphatidylcholine complex developed for drug delivery. Mol. Pharm..

[47] Zhang J., Tang Q., Xu X., Li N. (2013). Development and evaluation of a novel phytosome-loaded chitosan microsphere system for curcumin delivery. Int. J. Pharm..

[48] Minaei A., Sabzichi M., Ramezani F., Hamishehkar H., Samadi N. (2016). Co-delivery with nano-quercetin enhances doxorubicin-mediated cytotoxicity against MCF-7 cells. Mol. Biol. Rep..

[49] Nazari M., Ghanbarzadeh B., Kafil H.S., Zeinali M., Hamishehkar H. (2019). Garlic essential oil nanophytosomes as a natural food preservative: Its application in yogurt as food model. Colloids Interface Sci. Commun..

[50] Chanamai R., McClements D. (2002). Comparison of gum arabic, modified starch, and whey protein isolate as emulsifiers: Influence of pH, CaCl_2_ and temperature. J. Food Sci..

[51] Ansari F., Sobhani A., Salavati-Niasari M. (2018). Simple sol-gel synthesis and characterization of new CoTiO_3_/CoFe_2_O_4_ nanocomposite by using liquid glucose, maltose and starch as fuel, capping and reducing agents. J. Colloid Interface Sci..

[52] Papoutsis K., Golding J.B., Vuong Q., Pristijono P., Stathopoulos C.E., Scarlett C.J., Bowyer M. (2018). Encapsulation of citrus by-product extracts by spray-drying and freeze-drying using combinations of maltodextrin with soybean protein and ι-Carrageenan. Foods.

[53] Gangurde A.B., Ali M.T., Pawar J.N., Amin P.D. (2017). Encapsulation of vitamin E acetate to convert oil to powder microcapsule using different starch derivatives. J. Pharm. Investig..

[54] Cortes U.A.B., Gutiérrez M.C., Mendoza D.G., Salitre L.G., Vargas A.S., Catzim C.E.A., Durán C.C., Valenzuela B.E.L. (2021). Microencapsulation and antimicrobial activity of extract acetone-methanol of *Hibiscus sabdariffa* L. using a blend modified starch and pectin as a wall material. Ind. Crops Prod..

[55] Clogston J.D., Patri A.K., Scott E.M. (2011). Zeta potential measurement. Characterization of Nanoparticles Intended for Drug Delivery.

[56] Bhattacharjee S. (2016). DLS and zeta potential-what they are and what they are not?. J. Control. Release.

[57] Tang Q., Cao B., Wu H., Cheng G. (2013). Cholesterol-peptide hybrids to form liposome-like vesicles for gene delivery. PLoS ONE.

[58] Amit P., Tanwar Y., Rakesh S., Poojan P. (2013). Phytosome: Phytolipid drug delivery system for improving bioavailability of herbal drug. J. Pharm. Sci. Biosci. Res..

[59] Hindarto C.K., Surini S., Saputri F.C., Irawan C. (2017). In vivo evaluation of luteolin-loaded phytosome. J. Pharm. Innov..

[60] Ramteke K.H., Dighe P.A., Kharat A.R., Patil S.V. (2014). Mathematical models of drug dissolution: A review. Sch. Acad. J. Pharm..

[61] Siepmann J., Peppas N.A. (2011). Higuchi equation: Derivation, applications, use and misuse. Int. J. Pharm..

[62] Varga N., Turcsányi Á., Hornok V., Csapó E. (2019). Vitamin E-loaded PLA-and PLGA-based core-shell nanoparticles: Synthesis, structure optimization and controlled drug release. Pharmaceutics.

[63] Hamedi S., Koosha M. (2020). Designing a pH-responsive drug delivery system for the release of black-carrot anthocyanins loaded in halloysite nanotubes for cancer treatment. Appl. Clay Sci..

[64] Choudhary M.I., Shaikh M., tul-Wahab A., ur-Rahman A. (2020). In silico identification of potential inhibitors of key SARS-CoV-2 3CL hydrolase (Mpro) via molecular docking, MMGBSA predictive binding energy calculations, and molecular dynamics simulation. PLoS ONE.

[65] Pruchnik H., Bonarska-Kujawa D., Kleszczyńska H. (2014). Effect of chlorogenic acid on the phase transition in phospholipid and phospholipid/cholesterol membranes. J. Therm. Anal. Calorim..

[66] Cordeiro R.M. (2018). Reactive oxygen and nitrogen species at phospholipid bilayers: Peroxynitrous acid and its homolysis products. J. Phys. Chem. B..

