# Supplementary material for: Formulation of Nano/Micro-Carriers Loaded with an Enriched Extract of Coffee Silverskin: Physicochemical Properties, In Vitro Release Mechanism and In Silico Molecular Modeling
Source: Pharmaceutics. 2022 Jan 4;14(1):112. doi: 10.3390/pharmaceutics14010112 (PMC8781543; doi:10.3390/pharmaceutics14010112)
Supplement: Supplementary file 1 [file pharmaceutics-14-00112-s001.zip › pharmaceutics-1477025-supplementary.pdf]

pharmaceutics

Supplementary Materials: Formulation of Nano/Micro-Carriers Loaded with an Enriched Extract of Coffee Silverskin: Physiochemical Properties, In Vitro Release Mechanism and In Silico Molecular Modeling

Faezeh Fathi, Samad N. Ebrahimi, João A. V. Prior, Susana M. L. Machado, Reza Mohsenian Kouchaksaraee, M. Beatriz P. P. Oliveira and Rita C. Alves

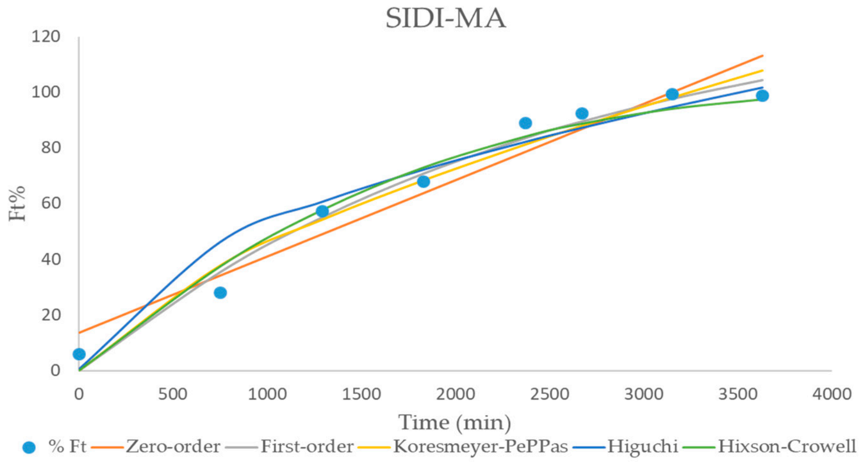

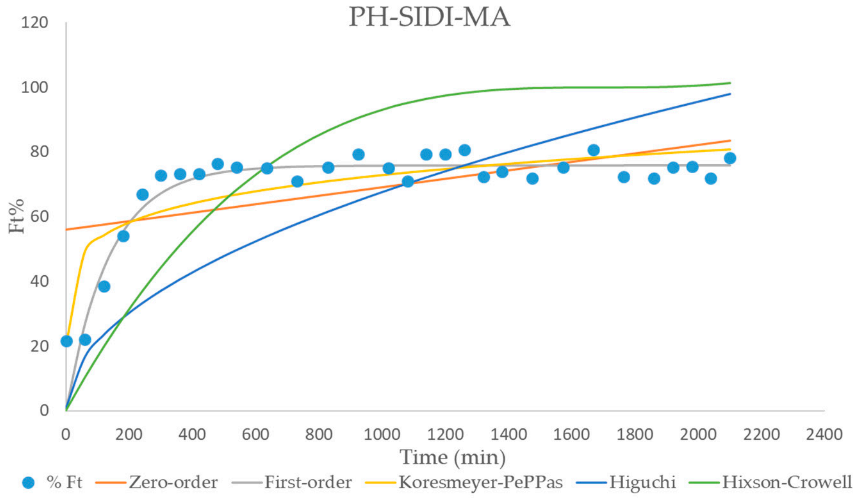

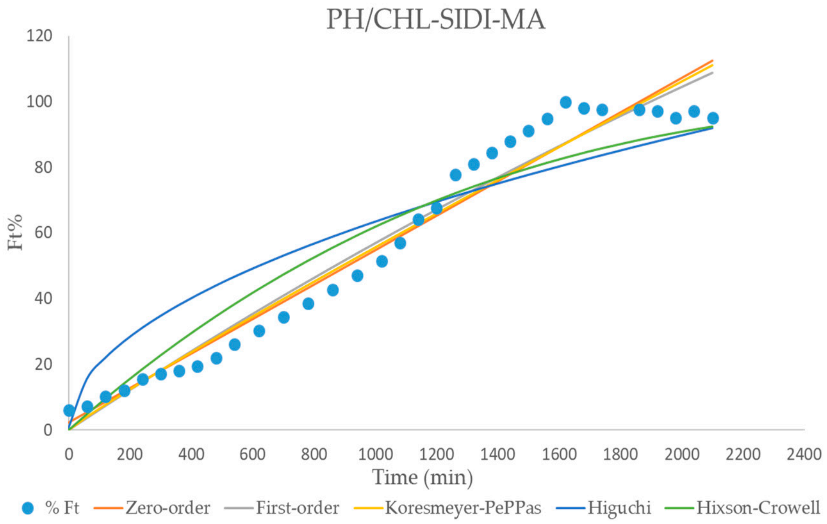

Figure S1. *In vitro* release assays curves associated to MA series at pH 2.1.

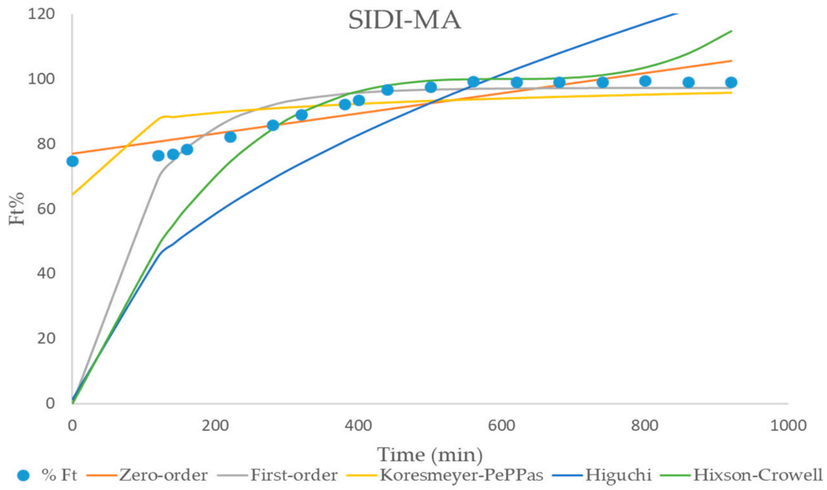

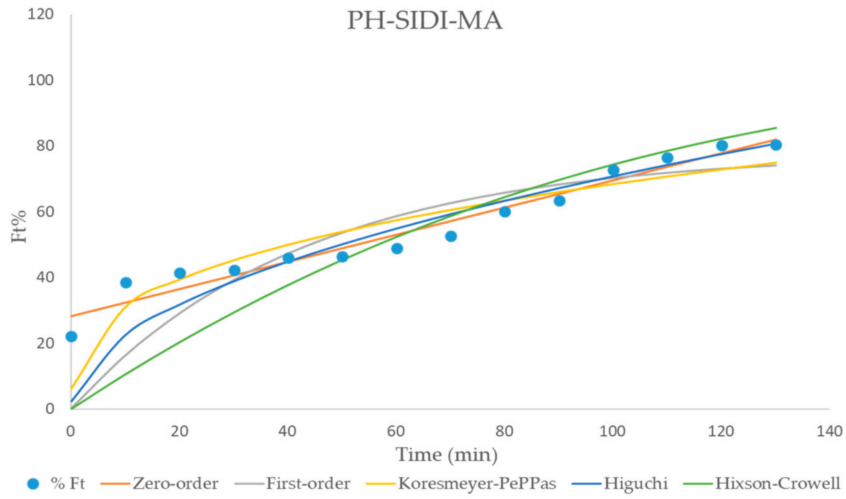

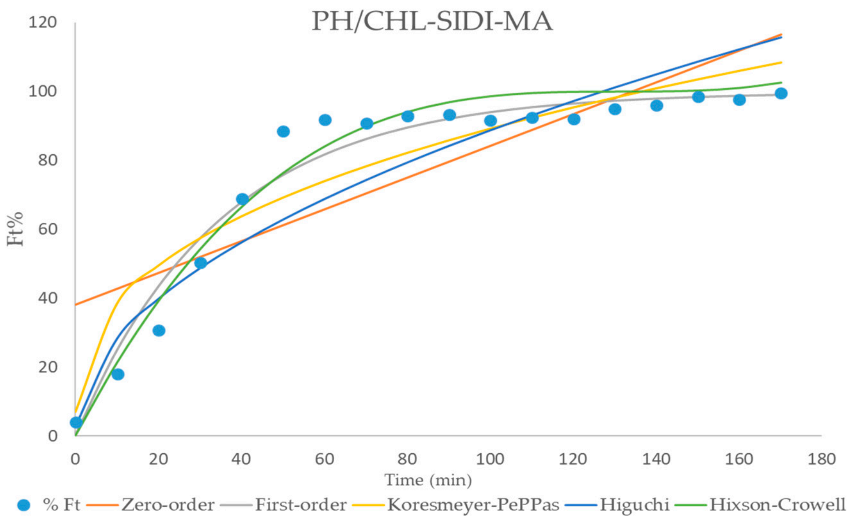

Figure S2. *In vitro* release assays curves associated to MA series at pH 7.8.

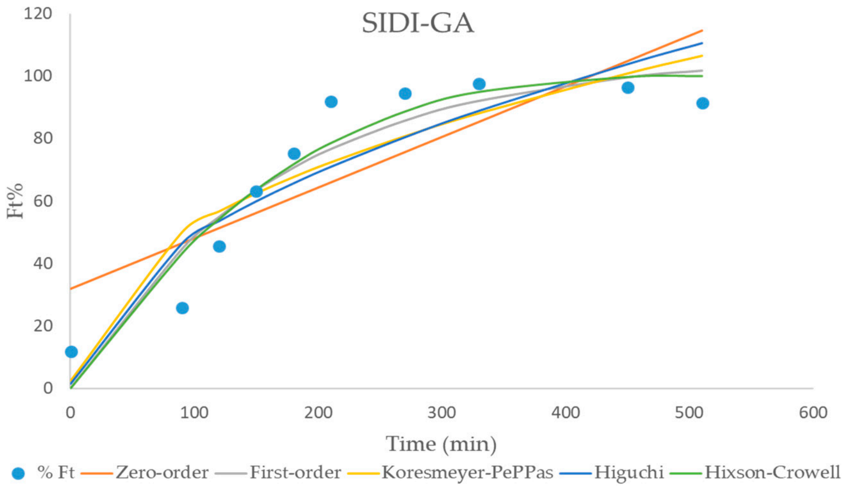

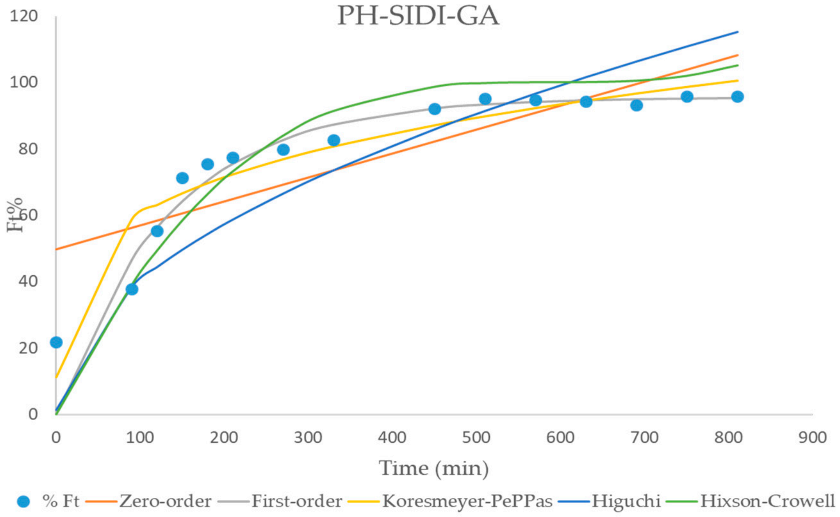

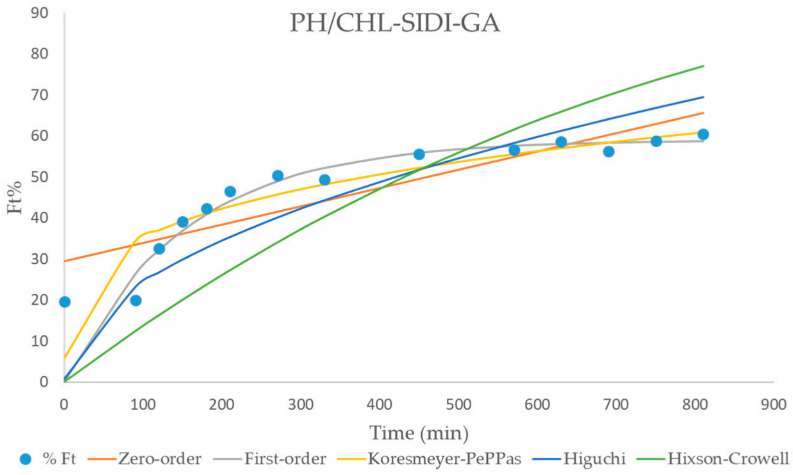

Figure S3. *In vitro* release assays curves associated to GA series at pH 2.1.

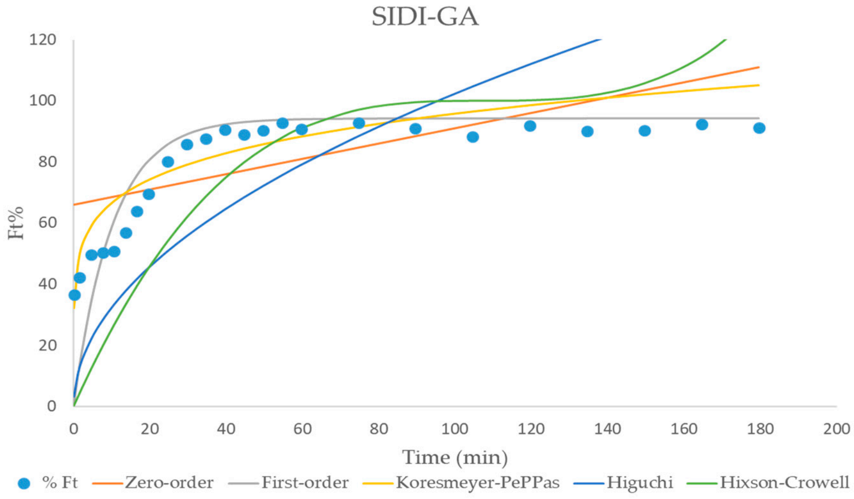

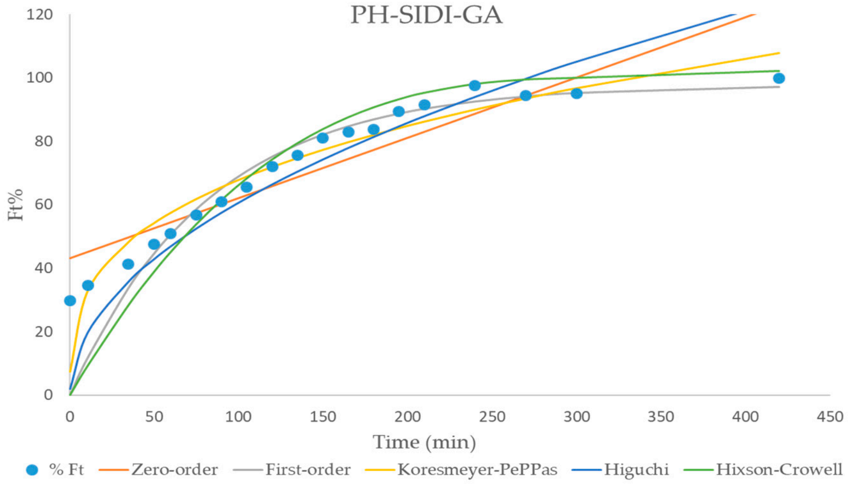

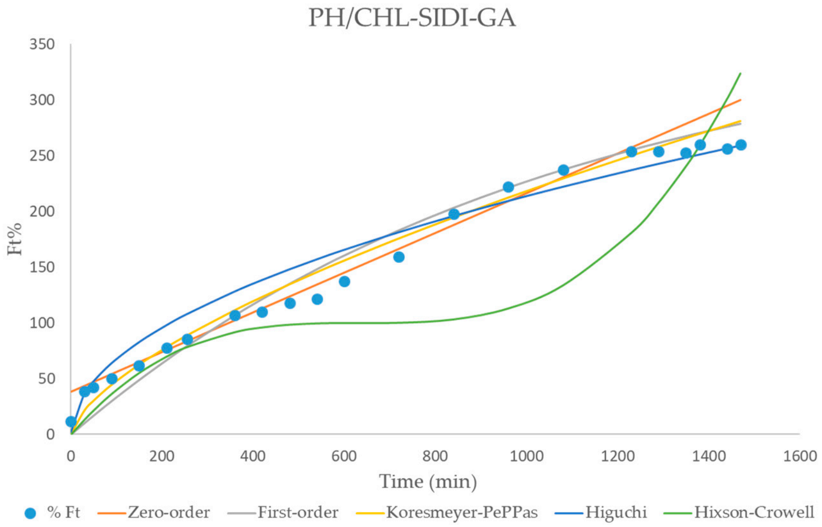

Figure S4. *In vitro* release assays curves associated to GA series at pH 7.8.

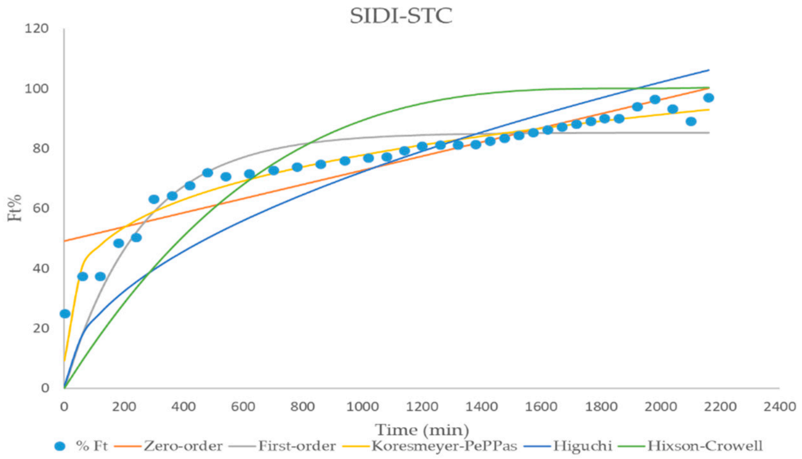

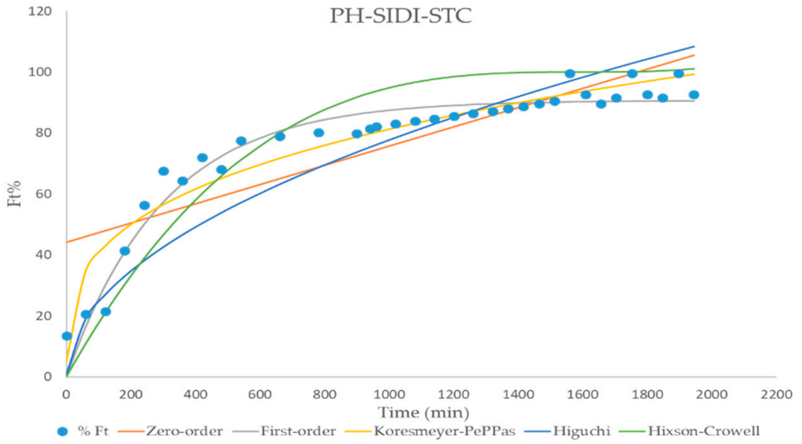

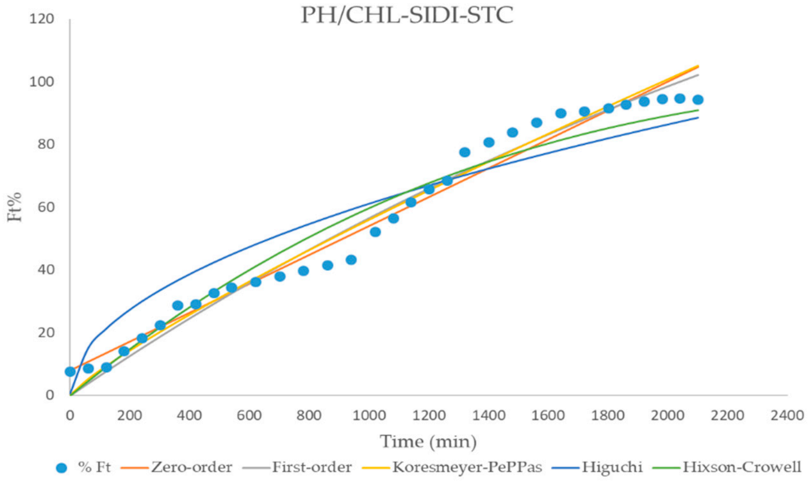

Figure S5. *In vitro* release assays curves associated to STC series at pH 2.1.

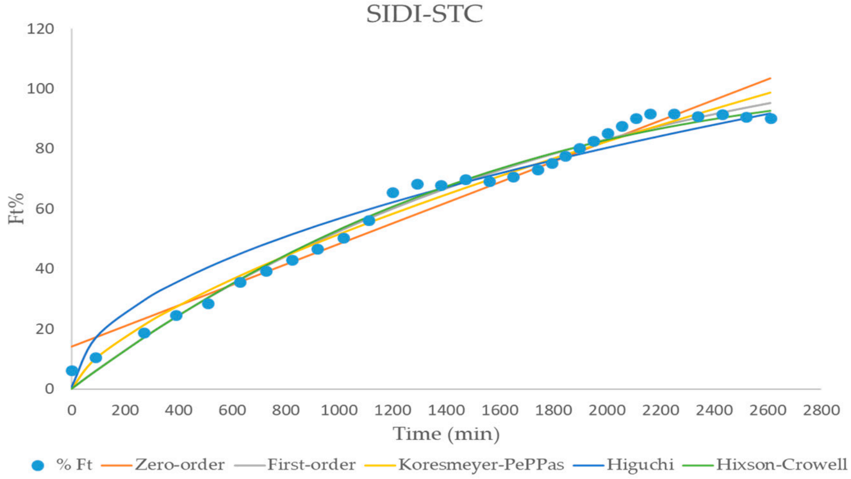

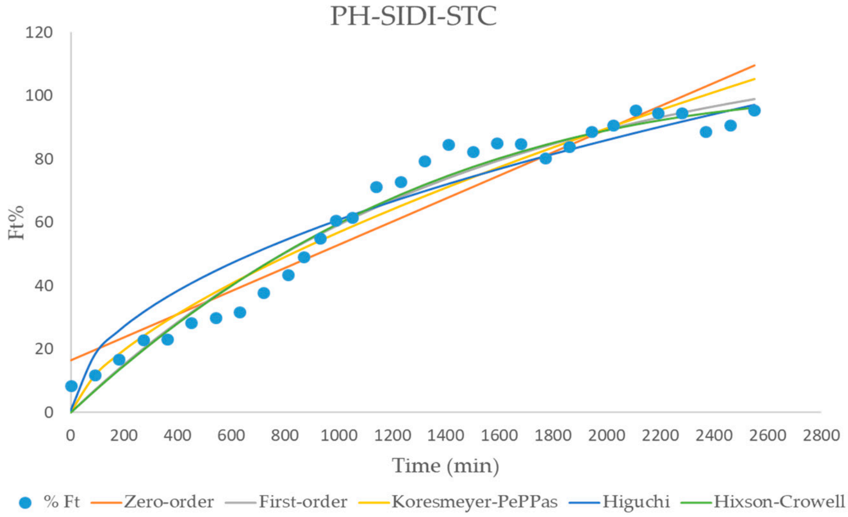

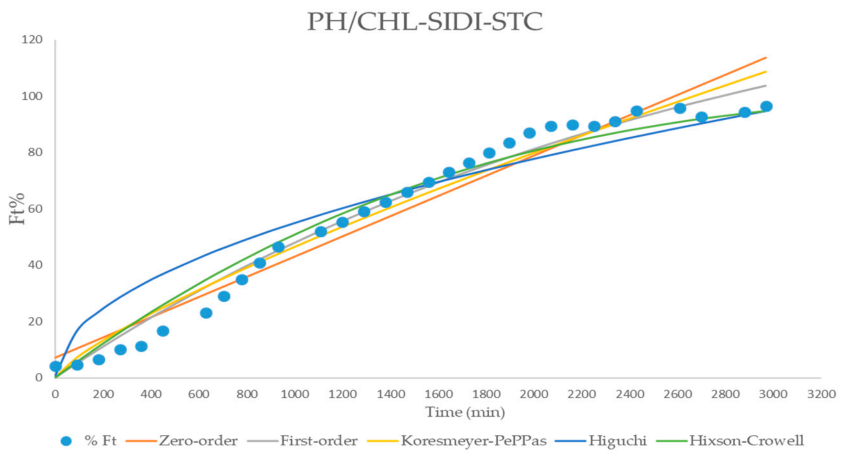

Figure S6. *In vitro* release assays curves associated to STC series at pH 7.8.

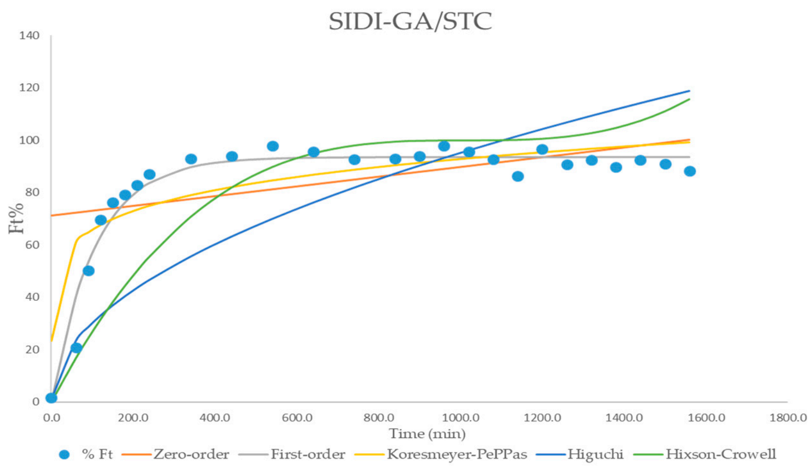

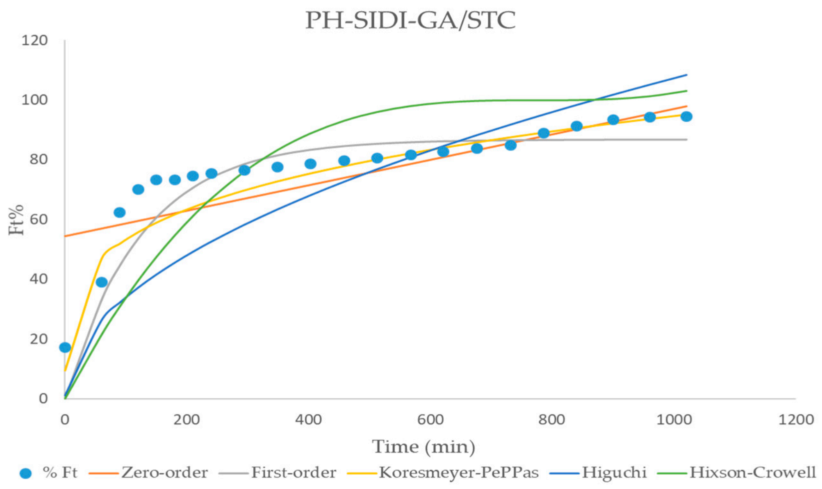

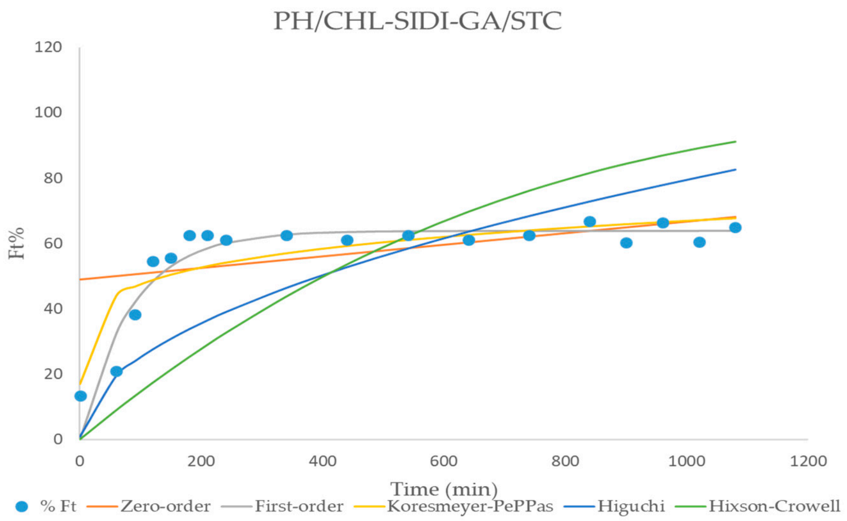

Figure S7. *In vitro* release assays curves associated to GA/STC series at pH 2.1.

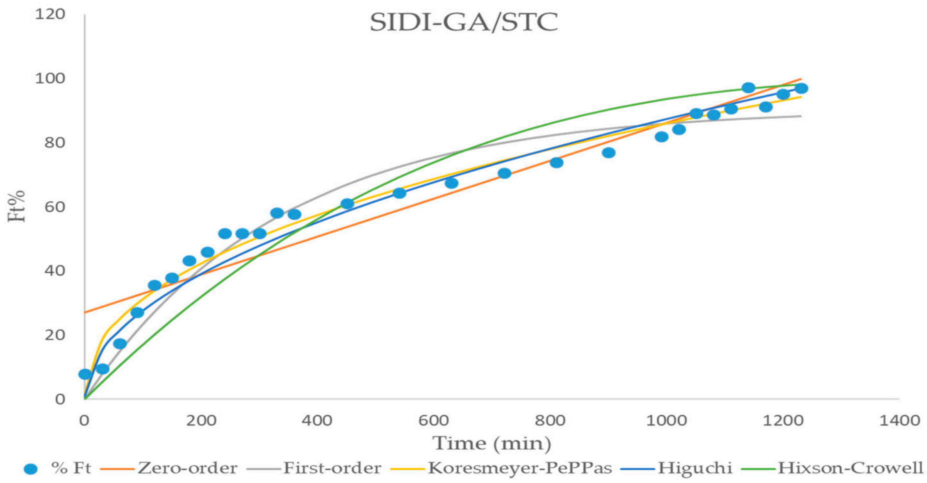

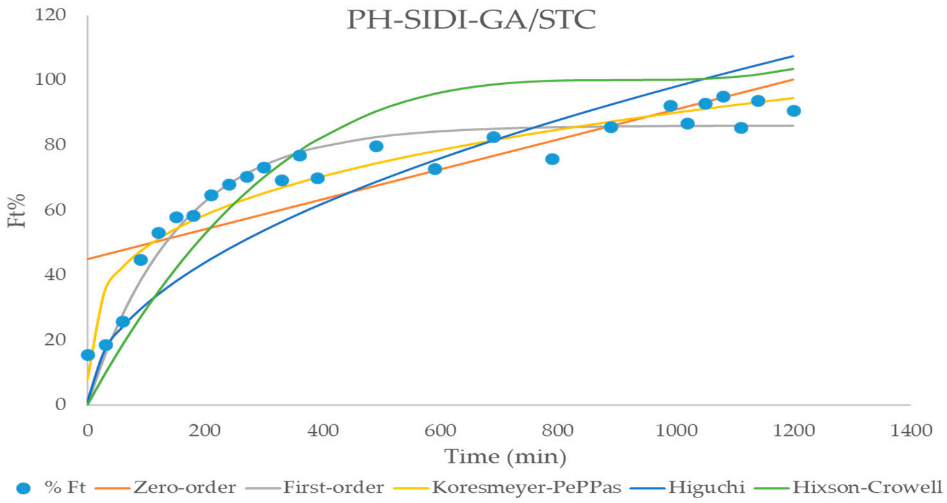

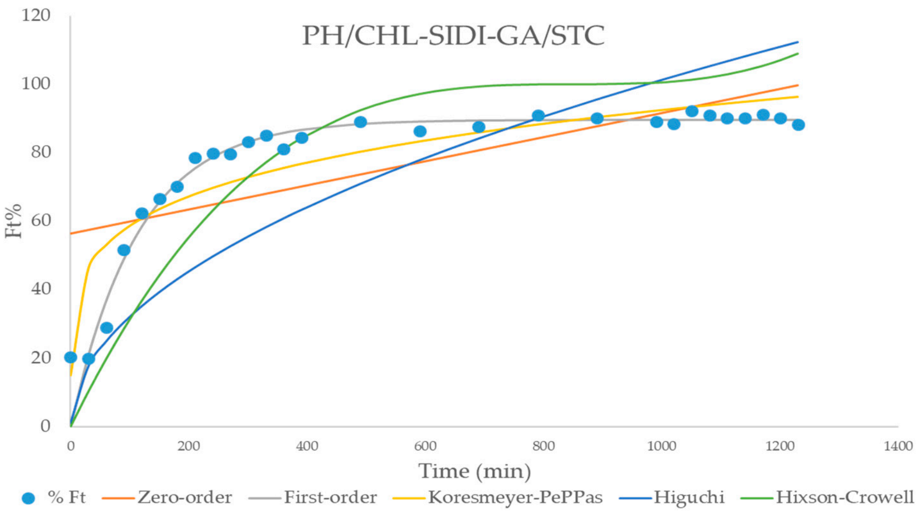

Figure S8. *In vitro* release assays curves associated to GA/STC series at pH 7.8.

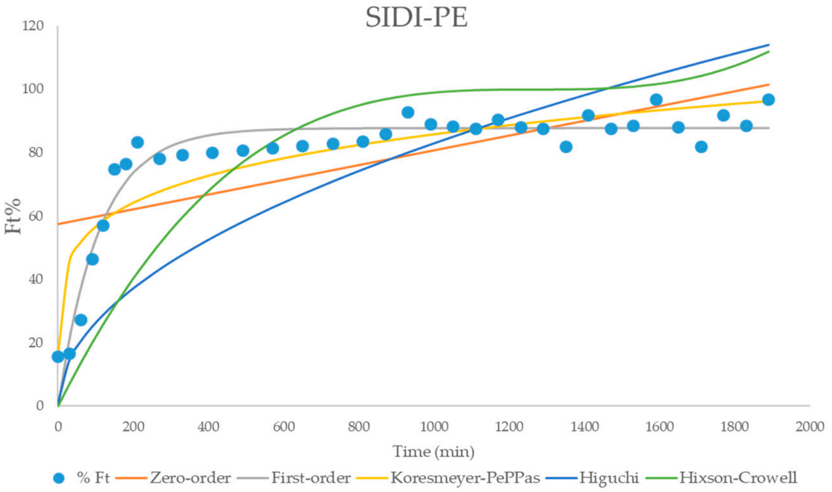

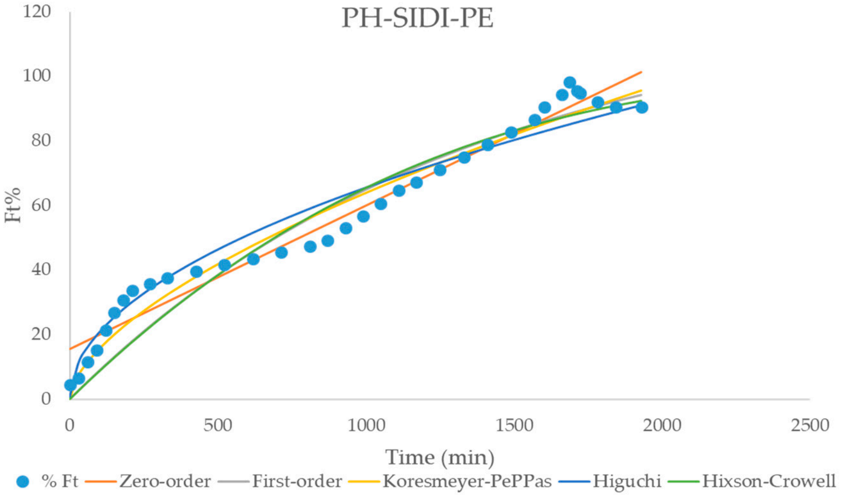

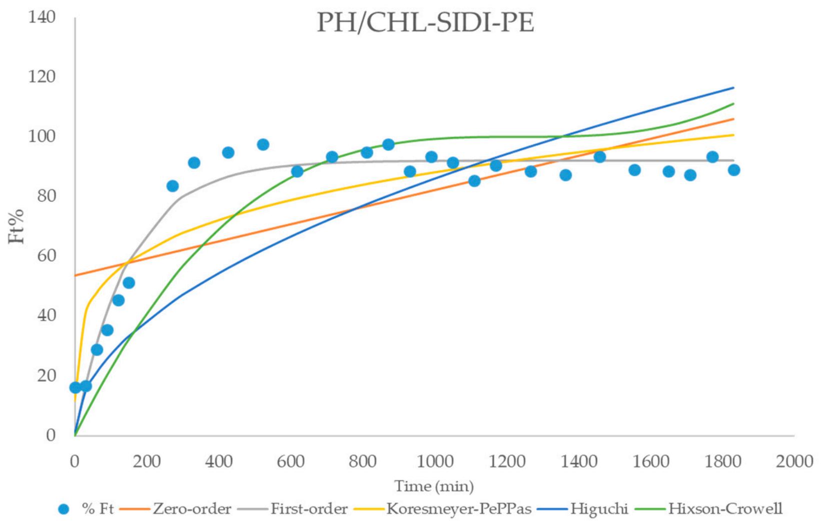

Figure S9. *In vitro* release assays curves associated to PE series at pH 2.1.

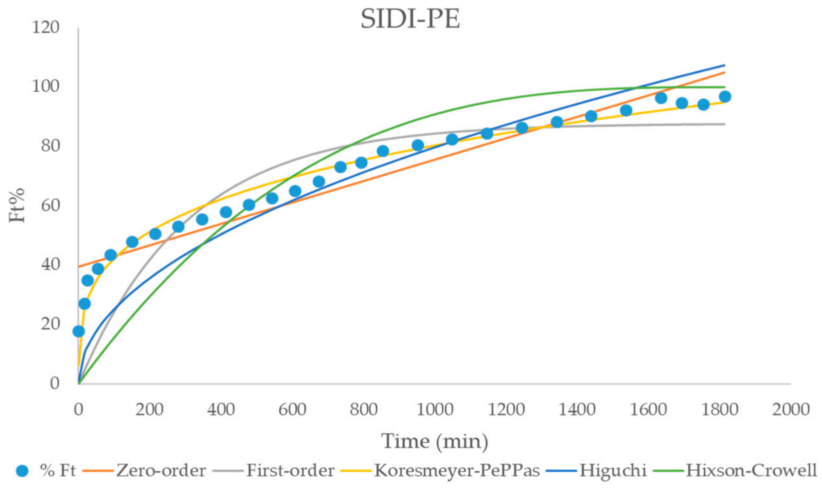

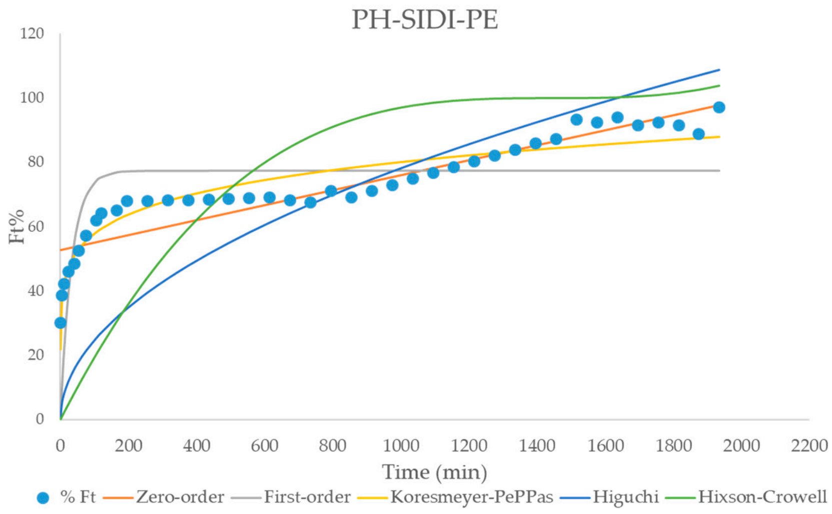

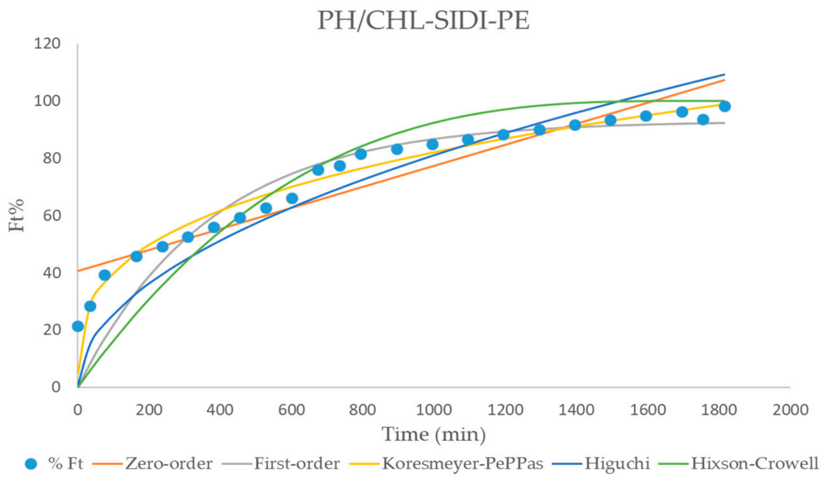

Figure S10. *In vitro* release assays curves associated to PE series at pH 7.8.
